# Supplementary material for: Characterization of intact mRNA-based therapeutics by charge detection mass spectrometry and mass photometry
Source: Mol Ther Methods Clin Dev. 2025 Mar 19;33(2):101454. doi: 10.1016/j.omtm.2025.101454 (PMC11999443; doi:10.1016/j.omtm.2025.101454)
Supplement: Document S1. Figures S1–S11 and Tables S1 and S2 [file mmc1.pdf]

OMTM, Volume *33*

Supplemental information

Characterization of intact mRNA-based therapeutics by charge detection mass spectrometry and mass photometry

Evolène Deslignière, Lauren F. Barnes, Thomas W. Powers, Olga V. Friese, and Albert J.R. Heck

Table S1. Experimental parameters used for CDMS experiments under native and denaturing conditions. The parameters are given for mRNA samples reported in Figure 2. Intensity to charge calibration factors were calculated as described in Wörner, T.P. *et al.*, *Nat Methods* **2020**, 17, 395–398. Differences in calibration factors account for data being recorded on two different UHMR instruments.

		**EPO mRNA**	**DS3 mRNA**	**DS1 mRNA**	**EPO mRNA**	**DS3 mRNA**	**DS1 mRNA**
		Native (low charges)			***Denaturing (high charges)***		
**MS parameters**	Polarity	Positive	Positive	Positive	Positive	Positive	Positive
	Capillary voltage (kV)	1.5	1.5	1.5	1.5	1.5	1.5
	Capillary temperature (°C)	250	250	250	250	250	250
	S-lens RF level (%)	200	200	200	200	200	200
	m/z range	5,000-15,000	10,000-25,000	26,700-50,000	2,000-7,000	3,000-10,000	2,500-10,000
	Ion injection time (ms)	5	30	500	2	1	10
	Transient time (ms)	512	256	128	128	128	128
	Source DC offset (V)	21	21	21	-	21	21
	In-source trapping	Desolvation -40<br>Trapping 10	Desolvation -60<br>Trapping 10	Desolvation -70<br>Trapping 10	-	-	-
	HCD voltage (V)	120	115	110	50	60	60
	In-source CID (eV)	20	10	-	-	-	-
	Trap gas setting	4	3.5	3.5	2	3	2
	UHV readout (1e-10 mbar)	2.1	1.6	1.6	1.1	2	0.6
	Collision gas	Nitrogen	Nitrogen	Nitrogen	Nitrogen	Nitrogen	Nitrogen
	Injection flatapole (V)	8	8	4	8	8	8
	Inter-flatapole lens (V)	7	7	4	7	7	7
	Bent flatapole (V)	6	6	6	6	6	6
	Ion transfer target	High *m/z*	High *m/z*	High *m/z*	High *m/z*	High *m/z*	High *m/z*
	Detector optimization	High *m/z*	High *m/z*	High *m/z*	High *m/z*	High *m/z*	High *m/z*
	Intensity to charge calibration factor	12.52	14.07	14.07	14.07	12.52	14.07

Table S2. Overview of the experimental mRNA masses obtained in MP using a mRNA- *vs.* protein-based calibration. For mRNA data, each mass was calculated by building a new calibration curve that did not include the measured mRNA (i.e., by successively considering each mRNA as an unknown sample).

Sample	mRNA-based calibration		Protein-based calibration	
	Experimental mass (kDa)	Deviation from theory (%)	Experimental mass (kDa)	Deviation from theory (%)
**EPO 5moU**	281 ± 56	-0.7	256 ± 50	-9.5
**EGFP**	329 ± 54	1.7	290 ± 50	-10.4
**Cre 5moU**	439 ± 55	-1.2	399 ± 49	-10.2
**OVA**	459 ± 56	-1.7	418 ± 51	-10.5
**Fluc**	624 ± 67	0.3	577 ± 58	-7.3
**β-Gal**	1092 ± 70	-1.3	998 ± 67	-9.8

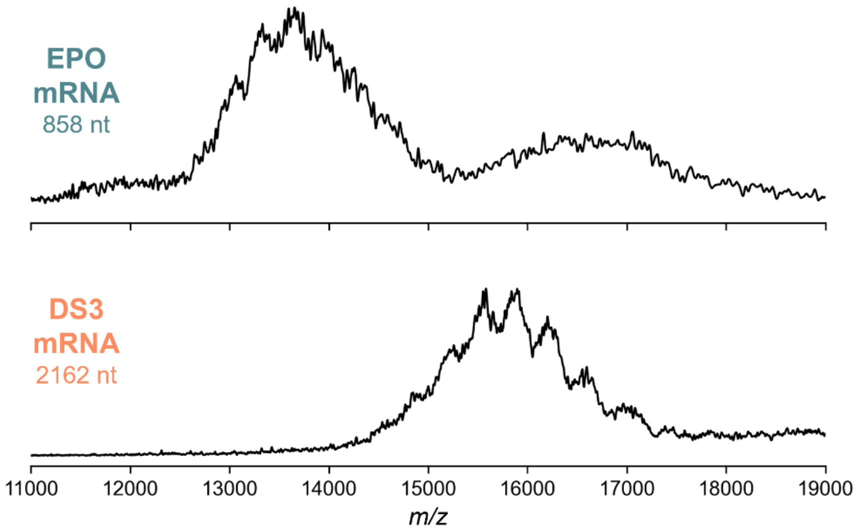

Figure S1. Native mass spectra of mRNAs recorded in negative ESI ion mode. Mass profiles exhibit more adducts than spectra acquired in positive ionization mode, making charge assignment and thus mass determination more difficult.

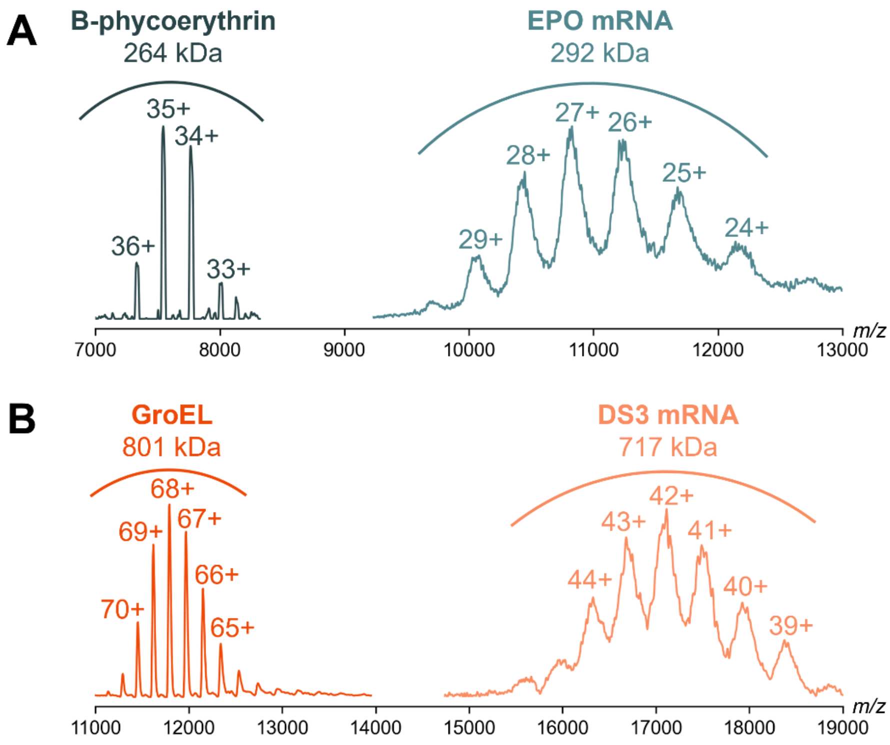

Figure S2. Native mass spectra of mRNA *vs.* proteins of similar sizes. Comparisons between **(A)** the protein complex B-phycoerythrin and EPO mRNA, and **(B)** the GroEL protein complex and DS3 mRNA. Spectra were recorded in positive ion mode and from 150 mM aqueous ammonium acetate solution. Note that mRNAs were sprayed by adding 50% methanol for better ionization efficiency.

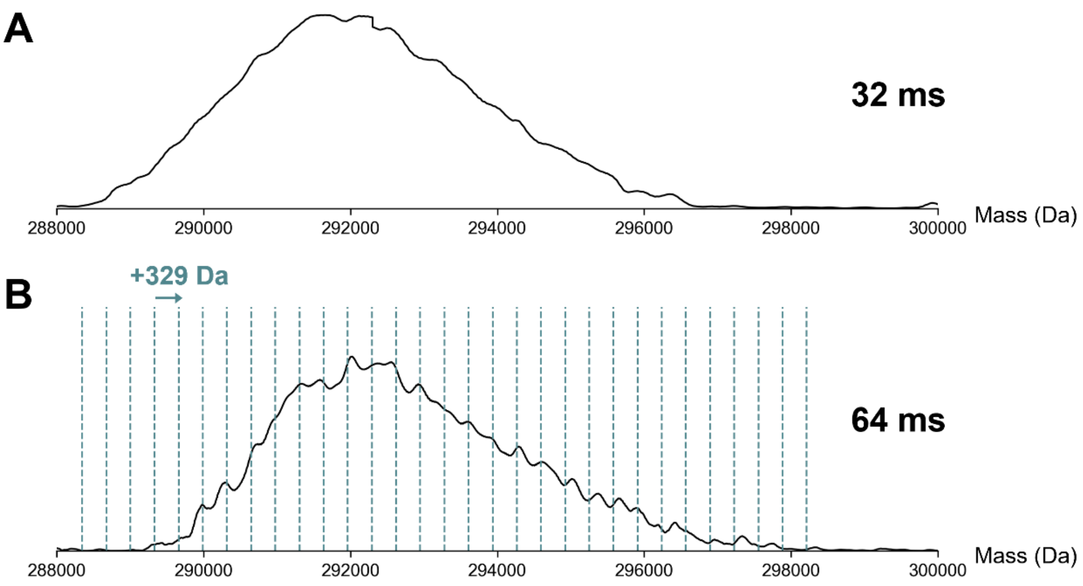

Figure S3. Mass deconvolution of EPO mRNA. Native mass spectra were recorded at transient lengths of **(A)** 32 ms and **(B)** 64 ms. The broad heterogeneous mass distribution is mostly due to the presence of poly(A) tail variants, separated by +329 Da (adenosine) as seen at extended transient length.

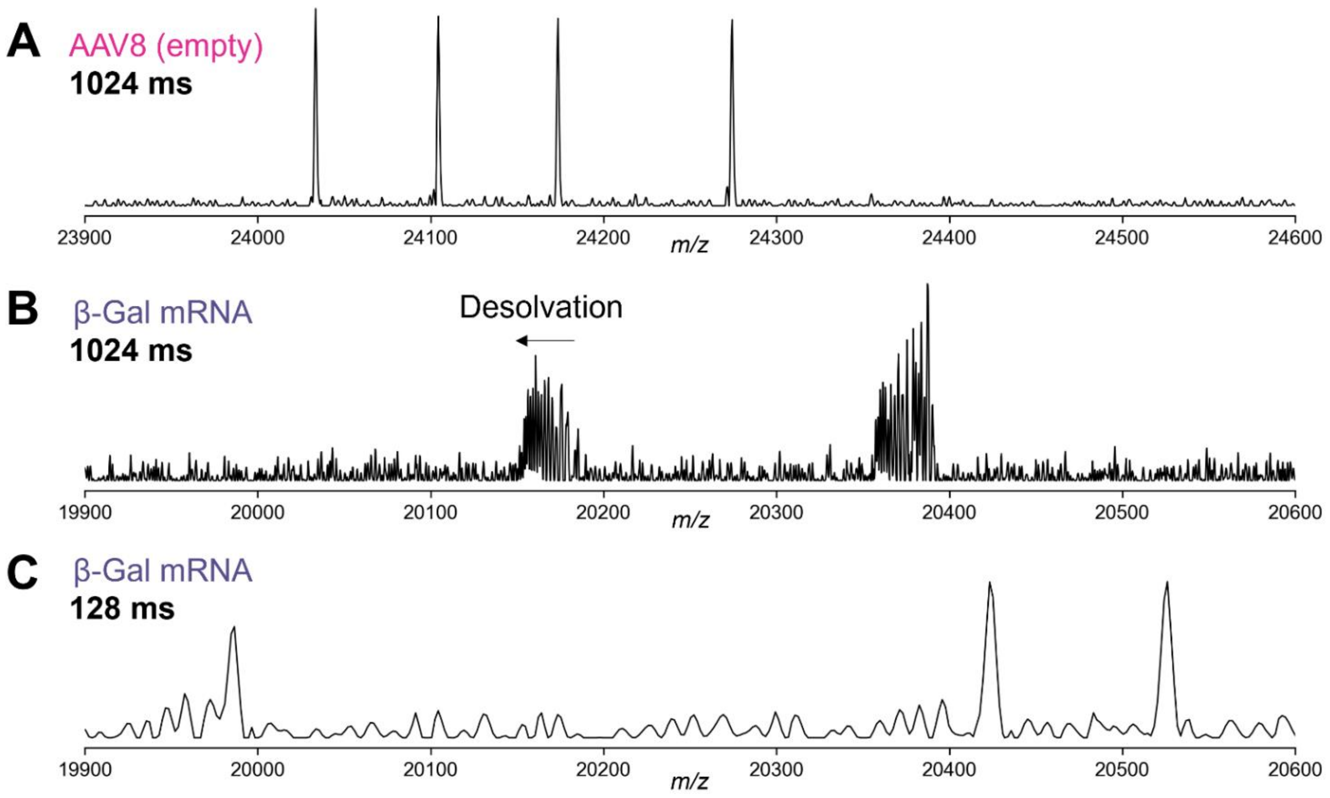

Figure S4. Non-ideal behaviors of mRNA single ions chased by CDMS, under native conditions. (A) In CDMS, single ions from an empty adeno-associated viral capsid (i.e. made up from proteins) are stable for 1 s. **(B)** In contrast, ions from the β-Gal mRNA show non-ideal behaviors over the 1 s-transient. Ions are drifting in *m/z* due to extensive desolvation. **(C)** By recording in CDMS shorter transients (128 ms) for the β-Gal mRNA, the ion drift is somewhat reduced, but can still occur (e.g. the ion at ~20,000 *m/z* has a lower intensity than expected due to neutral losses leading to peak splitting). Shorter transients result in a much lower resolution.

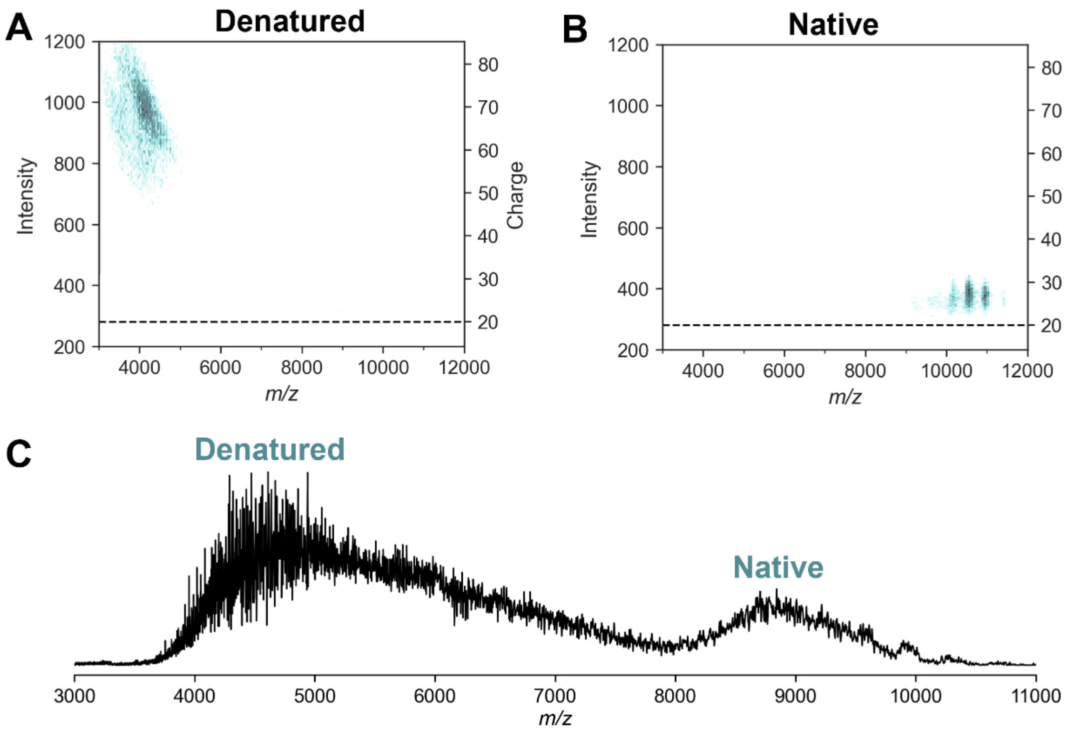

Figure S5. Native *vs.* denatured CDMS data for EPO mRNA. (A) Two-dimensional CDMS histogram of single ions in denaturing conditions (128 ms). The noise band is represented as a dotted line. **(B)** Two-dimensional CDMS histogram of native ions (512 ms). **(C)** Example of mass spectrum showing partial denaturation of the mRNA using methanol (1:3 v/v). MS parameters were tuned specifically to observe both populations simultaneously, and thus differ from those used in panels A and B.

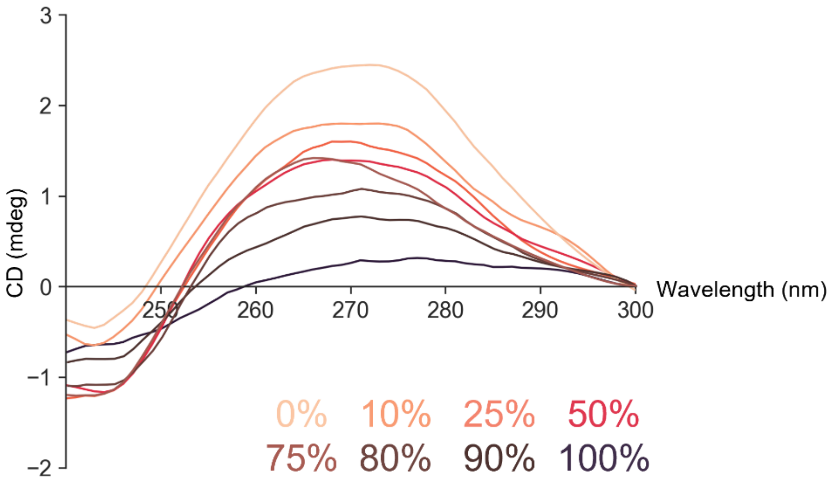

Figure S6. Circular dichroism spectra of the Cre mRNA, in water diluted with increasing percentages of methanol. Measurements were recorded in triplicate on a Jasco J-810 spectropolarimeter, using steps of 1 nm. For each solvent condition, the solvent-only spectrum (blank) was subtracted to the analyte measurement.

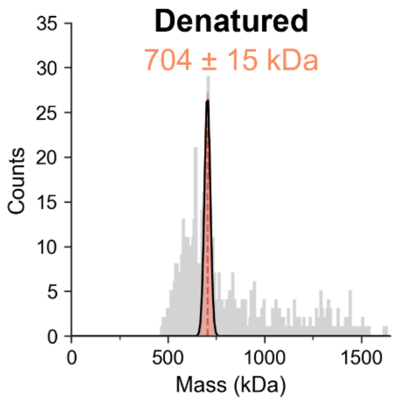

Figure S7. Mass histogram obtained by CDMS for the denatured DS3 mRNA using a transient length of 24 s. Extended transient lengths were recorded as reported in Deslignière, E. *et al.*, *Nat Methods* **2024**, 21, 619–622. The experimental mass at 24 s (704 kDa) is closer to the theory (702 kDa) than after 128 ms (743 kDa, Figure 2). This suggests that solvent molecules and labile adducts have been removed after prolonged trapping, and were contributing to the mass excess at short transients. Single ions undergo more collisions with neutral gas molecules as the transient time increases, with the mRNA eventually reaching full desolvation.

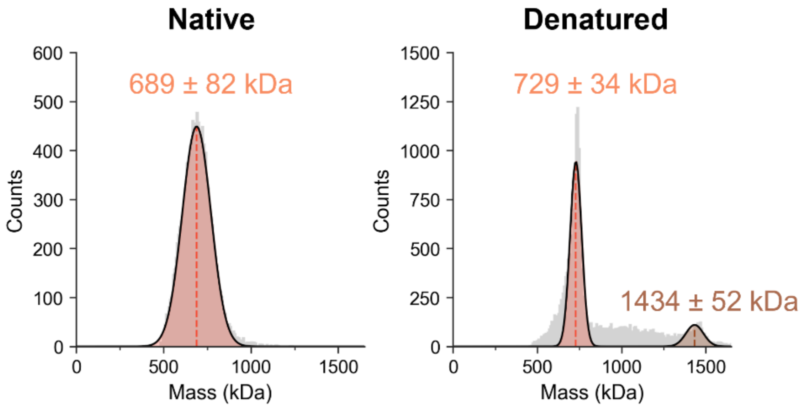

Figure S8. Mass histograms obtained by CDMS for the DS3 mRNA using isopropanol (1:3 v/v) instead of methanol to induce partial mRNA denaturation. Data were extracted from lowly charged species (37-50+, native) or highly charged populations (100-200+, denatured).

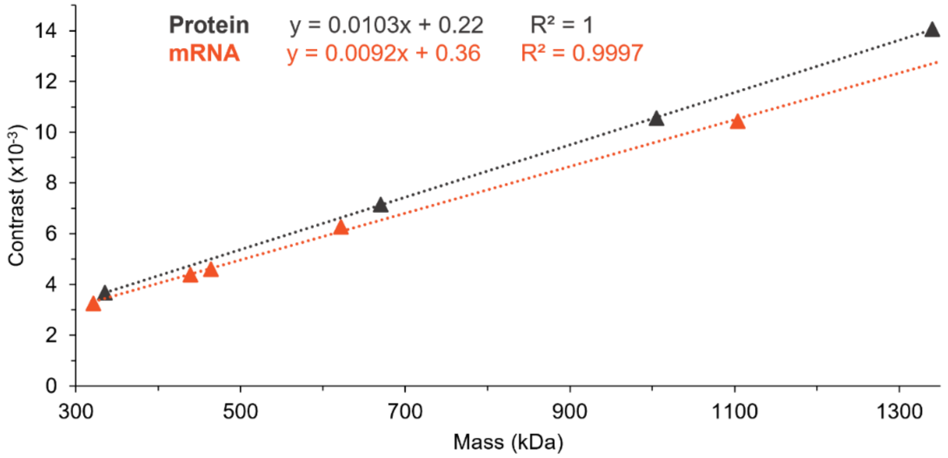

Figure S9. MP calibration curves using proteins (grey) or mRNAs (orange). The protein calibration is based on thyroglobulin multimers. The mRNA calibration contains EGFP, Cre, OVA, Fluc and β-Gal mRNAs. The differences in calibration curves show that the contrast highly depends on the nature of the analyte, with in particular large differences in between protein assemblies and mRNA.

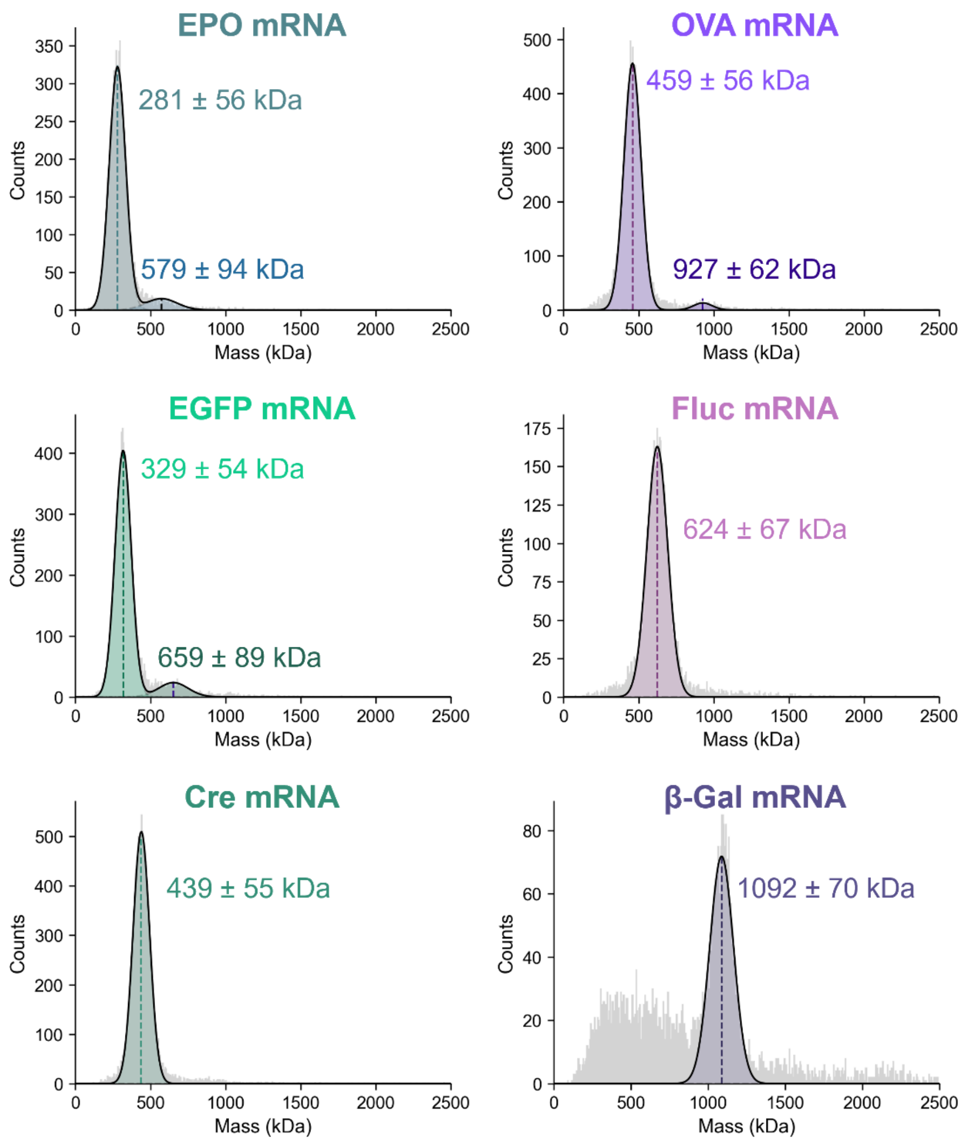

Figure S10. MP analyses of mRNAs. For all mRNAs, the mass of the main monomeric species is within 2% of the expected theory (see Table S2). Small amounts of dimeric populations are detected in EPO, OVA, and EGFP mRNAs. Many LMWS are clearly detected in the β-Gal sample, on the left of the main peak. Because the mass photometer used here is not adapted for the detection of LMWS < 250 kDa, it is likely that fragments are overlooked in other samples.

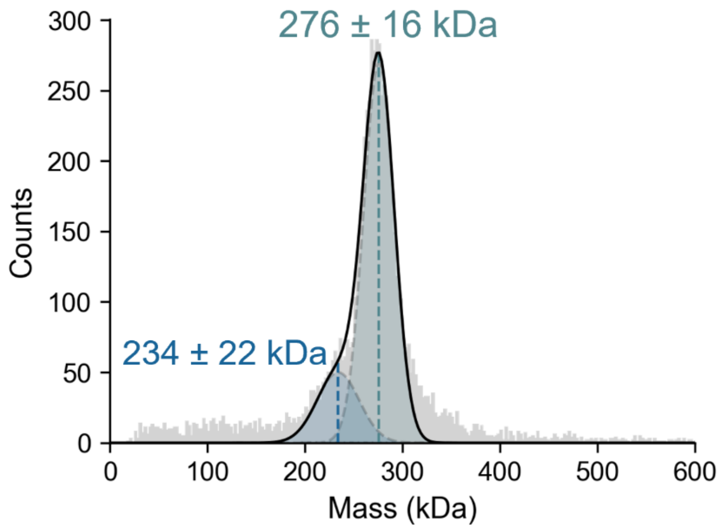

Figure S11. MP analysis of the EPO mRNA sample on a Two^{MP} mass photometer. This instrument is better adapted for the detection of LMWS < 250 kDa compared to the Samux^{MP}, the instrument used for the data described in the main manuscript. The Two^{MP} has a higher resolution, allowing to observe a left-side shoulder species of 234 kDa. Of note, dimeric EPO is not detected compared to what is seen on the Samux^{MP} (see Figure S10), which might be due to different glass slides coating used for the different experiments.
