# Supplementary material for: Characterization of intact mRNA-based therapeutics by charge detection mass spectrometry and mass photometry
Source: Mol Ther Methods Clin Dev. 2025 Mar 19;33(2):101454. doi: 10.1016/j.omtm.2025.101454 (PMC11999443; doi:10.1016/j.omtm.2025.101454)
Supplement: Document S2. Article plus supplemental information [file mmc2.pdf]

Molecular Therapy
Methods & Clinical Development
Original Article

Characterization of intact mRNA-based therapeutics by charge detection mass spectrometry and mass photometry

Evolène Deslignière,[1,2] Lauren F. Barnes,[3] Thomas W. Powers,[3] Olga V. Friese,[3] and Albert J.R. Heck[1,2]

[1]Biomolecular Mass Spectrometry and Proteomics, Bijvoet Centre for Biomolecular Research and Utrecht Institute for Pharmaceutical Sciences, University of Utrecht, 3584 CH Utrecht, the Netherlands; [2]Netherlands Proteomics Center, 3584 CH Utrecht, the Netherlands; [3]BioTherapeutics Pharmaceutical Sciences, Pfizer Inc, Chesterfield, MO 63017, USA

The impressive success of mRNA-based vaccines to combat COVID-19 has encouraged biopharmaceutical companies to invest in broader applications of alike vaccines for various diseases. Analytical approaches must keep pace to support this surge in the development of mRNA-based therapies. Intact mass analysis of mid- to large mRNA molecules (>1,000 nt) poses significant analytical challenges due to mRNA size, heterogeneity, and instability. Here, we demonstrate how single-particle Orbitrap-based charge detection mass spectrometry (CDMS) and mass photometry (MP) approaches can rapidly measure the mass of various intact high-mass capped mRNAs, up to 9,400 nt (∼3 MDa) in size. While ensemble MS yielded approximate masses for mRNAs <2,000 nt, it failed to provide information on samples of longer sequences. The drawbacks of ensemble MS could be avoided by recording individual ions. Low-charge mRNA components showed unstable ion behavior, hampering initial CDMS measurements, whereas high-charge populations offered better signal-to-noise and reduced charge uncertainty, with drastically improved mass accuracy. Lastly, in-solution MP enabled the measurement of mRNAs with high accuracy, while revealing low amounts of mRNA fragments and dimers that are sometimes overlooked in CDMS. Overall, CDMS and MP provide complementary methods that enable the study of large heterogeneous mRNA without requiring prior digestion or online separation.

INTRODUCTION

Following the COVID-19 pandemic and the regulatory approval of two highly efficient mRNA-based vaccines encoding the SARS-CoV-2 spike protein, messenger RNA (mRNA)-based technology has emerged as a promising alternative to conventional vaccines against infectious diseases, fostering the research and development of new mRNA therapeutics. Work on mRNA vaccines is not restricted to COVID-19, with clinical trials targeting influenza,[1] HIV,[2] cancers,[3–5] and more rare diseases.[6] A new mRNA-based vaccine against respiratory syncytial virus has recently been approved by the US Food and Drug Administration. The number of mRNA drugs in the clinical pipeline worldwide is rapidly growing, with several hundred mRNA products under development.[7] One of the main benefits of the mRNA technology is that it can be tailored rapidly to different diseases or variants. In addition, mRNA vaccines can be manufactured in a cell-free manner, allowing fast and scalable production.[8] Due to their numerous advantages, mRNAs are soon expected to become a major class of therapeutics.[9] To support the surge in the development of mRNA therapies, analytical approaches must keep pace to ensure the identity, stability, structural integrity, safety, and efficacy of evolving therapeutic mRNA candidates.

Considerations for mRNA testing are quite different than they are for chemically synthesized oligonucleotides. *In vitro* transcription (IVT) creates mRNA from plasmid DNA templates in a one-pot enzymatic reaction, which adds complexity to mRNA characterization. Indeed, the reaction mixture contains not only the desired mRNA product but also salts, nucleotide triphosphates, enzymes, remaining DNA plasmids, and unwanted mRNA by-products (e.g., double-stranded RNA, fragments, aggregates).[10] These impurities are removed by mRNA purification. mRNA typically contains five structural features[11]: the 5′ cap structure, the 5′ and 3′ untranslated regions, an open reading frame, and a tail of adenosine residues (poly(A) tail) that enhances mRNA stability and translation. Proper capping of the 5′ terminus and appropriate length of the poly(A) tail are essential for mRNA translation.[12] The alteration of any of these structural features during purification, manufacturing, or storage can reduce mRNA safety and efficiency.[13] Premature transcriptional termination or hydrolysis can generate mRNA fragments, but such species generally lack translational viability.[14] The presence of double-stranded RNA (dsRNA) can induce severe immune responses,[15,16] with up-regulation of pro-inflammatory cytokines and cell death. This highlights the need to explore analytical strategies to confirm the identity, integrity, and purity of the mRNA product.

Received 24 September 2024; accepted 16 March 2025;
https://doi.org/10.1016/j.omtm.2025.101454.

Correspondence: Albert J.R. Heck, Biomolecular Mass Spectrometry and Proteomics, Bijvoet Centre for Biomolecular Research and Utrecht Institute for Pharmaceutical Sciences, University of Utrecht, 3584 CH Utrecht, the Netherlands.
E-mail: a.j.r.heck@uu.nl

Published by Elsevier Inc. on behalf of The American Society of Gene and Cell Therapy.
This is an open access article under the CC BY license (http://creativecommons.org/licenses/by/4.0/).

Currently, there are limited tools available to characterize mid- to large mRNA-based therapeutics, especially for mRNA above 2,000 nt.[17] Several chromatographic approaches have been employed to investigate mRNA impurities such as aggregates and fragments.[18] The analysis of RNAs between 1,000 and 8,000 nt by ion pair reversed-phase (IP-RP) has been achieved using different stationary phases.[19,20] Size-exclusion chromatography (SEC) has recently been explored to assess the nature of aggregates (covalent vs. non-covalent) based on SEC profiles obtained after heat treatment[21] or under denaturing vs. native conditions.[22] Different studies have evaluated the influence of the chromatographic conditions (e.g., pore size, mobile phase composition, column temperature).[21,23] D'Atri et al. recently developed a fit-for-all SEC method to characterize a series of commercial mRNAs up to 4,521 nt, even showing the potential of short columns for rapid mRNA analysis.[24] In addition to chromatography, capillary electrophoresis is also well established for assessing mRNA purity and integrity. Microchip capillary electrophoresis has been proposed as a fast method for RNA up to 6,000 nt.[25]

Only a few mass spectrometry (MS) techniques have been described for the characterization of large mRNAs. Different methods relying on enzymatic digestion, followed by IP-RP separation and tandem MS, have been developed for the sequence mapping of large mRNA, also allowing the assessment of the degree of capped and uncapped 5′ termini, and the microheterogeneity of the poly(A) tail.[26–28] Gau et al. reported a 56% sequence coverage based on unique fragments (100% including sequence isomers) using RNAse T_1 digestion for an mRNA vaccine with a length of 4,283 nt.[29] Such digestion-based approaches allow the confirmation of the mRNA identity but remain time-consuming and require extensive manual data analysis.

Bottom-up techniques are particularly well adapted to look at individual features (e.g., 5′ cap or adenosine tail); however, one way to monitor the integrity of the whole mRNA, and also the presence of potential impurities such as fragments and dsRNA, is to determine the molecular mass of intact mRNA. As mRNA therapeutics are typically large and heterogeneous, most conventional MS approaches fail to provide mass information at the intact level. However, by coupling IP-RP to MS using a mobile phase composed of diisopropylethylamine titrated with hexafluoroisopropanol, Brophy et al. were able to analyze a human erythropoietin (EPO)-encoding mRNA of 858 nt, including its poly(A) tail.[30] Moreover, native MS has been used to measure the mass of an intact mRNA construct with (783 nt, 254 kDa) and without (683 nt, 221 kDa) poly(A) tail.[22] In that study, a higher heterogeneity of the mass profile was observed in the presence of the poly(A) tail. This factor of heterogeneity makes the native MS analysis of larger mRNAs increasingly difficult as the charge state distribution can become unresolvable, hampering mass determination. To address this challenge, charge detection MS (CDMS), which simultaneously measures the *m/z* ratio and charge of single particles instead of ion clouds, could be beneficial.[31,32] This approach allows determination of the mass of even very large and polydisperse biomolecules.[33,34] This has been successfully applied to the characterization of DNA,[35–37] but work on mRNA remains scarce and restricted to commercial samples.[30,38] As an alternative to MS approaches, mass photometry (MP) relies on the interferometric scattering technique to detect single molecules in solution. Although the mass resolution and accuracy of MP fall short of what is possible with MS-based techniques,[39] MP can be used to rapidly measure the mass of intact mRNA while ensuring a native environment. It also enables the quantification of by-products, which is of interest for forced degradation studies.[22]

In the present work, we report on the combination of native MS, Orbitrap-based CDMS, and MP approaches to characterize mRNA products up to 9,400 nt (~3 MDa). Being the most mainstream of these three techniques, native MS was used first despite its rare application to mRNA. We show that native MS can help in assessing the mass of mid-sized mRNAs, but it only yields broad unresolved MS profiles beyond 1,000 nt, which prompted us to move to CDMS. However, performing our usual Orbitrap-based CDMS workflow in native conditions proved to be challenging because of unstable ion trajectories of many of the studied single ions. Instead, we developed a strategy relying on denaturing conditions that permits the determination of the masses of mid- to large mRNAs with high accuracy and resolution. To our knowledge, this is the first reported application of Orbitrap-based CDMS to very large mRNAs, including non-commercial samples. To validate our CDMS data, we also used MP to measure mRNA and their minor by-products. Considering the growing interest in mRNA therapeutics, research and development pipelines would strongly benefit from approaches such as Orbitrap-based CDMS and MP, as described here.

RESULTS

Native MS of mRNA up to 1 MDa

We first intended to analyze mRNA using conventional ensemble native MS, as it has been described recently as a promising avenue for mass determination of mid-sized mRNA.[22] Major challenges for intact analysis lie in the adduct formation propensities of mRNA and inherent mRNA heterogeneity. Note that all data presented thereafter were recorded in positive electrospray ionization (ESI) mode, as mass spectra generated in negative ESI mode exhibited even more adducts (Figure S1). To avoid undesirable cation adducts coming from, for example, buffers supplemented with Mg^{2+}, we used low concentrations of ammonium acetate, with methanol as co-solvent to improve the ionization efficiency (see materials and methods). Other parameters that play a crucial role in the quality of the mass profile are the capillary position and shape of the capillary tip, which can be difficult to reproduce.[40] After fine-tuning of the spray and MS parameters, resolved charge state distributions could be observed for mRNA samples up to ~1 MDa (Figure 1). In positive ion mode, charge states were lower for mRNAs compared to proteins of similar masses (Figure S2) as a consequence of the negatively charged RNA backbone.

For the EPO mRNA sample (858 nt), a mass of 292 ± 5 kDa was obtained for the single-stranded mRNA (Figure 1A). Traces of dimeric species were also detected, in line with previous reports.[30] Although

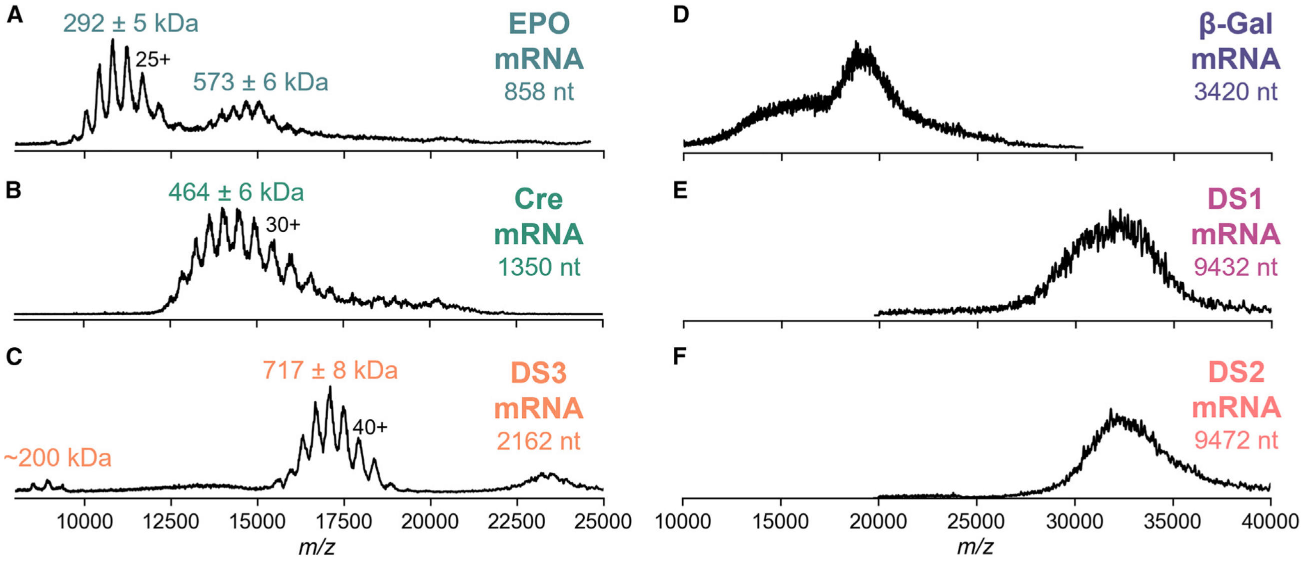

Figure 1. Native mass spectra in positive ESI mode of mid- to large mRNA samples
(A) EPO mRNA, (B) Cre mRNA, (C) DS3 mRNA, (D) β-Gal mRNA, (E) DS1 mRNA, and (F) DS2 mRNA. For EPO, Cre, and DS3 mRNAs, charges and masses could be assigned based on the resolved charge states, whereas the broad profiles seen for β-Gal, DS1, and DS2 hamper such a mass assignment by native MS.

masses could be extracted here, peaks were broad and heterogeneous, which is particularly obvious when compared to homogeneous proteins (Figure S2). We tentatively attribute this mRNA heterogeneity mostly to the presence of different lengths of poly(A) tail in the EPO mRNA product.[30,41] These variants, separated by ∼329 Da (adenosine), start becoming apparent at higher resolution (Figure S3). In addition, T7 RNA polymerase, used in IVT to catalyze mRNA formation, can introduce heterogeneity at both 5′ and 3′ ends.[42,43]

Moving up in size, experimental masses could also be determined for the Cre mRNA (464 ± 6 kDa) and the DS3 mRNA (717 ± 8 kDa), the latter showing minor populations corresponding to dimeric species and mRNA fragments (Figures 1B and 1C). The masses for both mRNA monomers were somewhat higher than expected, which may be attributed to counterions, similar to DNA measured with CDMS.[37] With a mixture of NH_4^+ and H^+ counterions coming from the use of the ammonium acetate solution, the experimental mass can exceed the theoretical mass, as we experimentally observe. More precisely, the phosphodiester backbone (pK_a ∼1) is fully ionized under these conditions (pH ∼7). However, the neutral ammonium acetate solution may undergo acidification in the ESI plume in positive ion mode,[44] meaning the phosphate backbone will remain negatively charged, but protonation of selected nucleobases can occur. Metallic cations (Na^+, K^+) and solvent molecules (due to incomplete desolvation) will also contribute to making the measured mass higher than the theoretical mass.

Lastly, for the three mRNA samples whose mass is larger than 1 MDa (∼1.1 MDa for β-galactosidase [β-Gal], ∼3 MDa for DS1 and DS2), only broad unresolved charge state distributions were observed, hampering any mass determination (Figures 1D–1F). At the intact level, these mRNA samples pose further challenges in terms of size, heterogeneity, desolvation, and transmission, impeding their analysis by traditional ensemble native MS.

Orbitrap-based CDMS under denaturing conditions allows accurate mass determination of even larger intact mRNA

To circumvent the limitations of ensemble native MS, we next moved to single-particle Orbitrap-based CDMS to enable the study of large mRNA at the intact level.

One of the primary challenges in a CDMS experiment is maintaining stable ion orbits within the Orbitrap mass analyzer to trace ions for extended transient lengths. While ensuring that ion stability for large proteins over long transients (512–1,024 ms) under native conditions is possible with optimized MS parameters,[45] we observed that intact mRNAs exhibit extensive non-ideal ion behavior when increasing the transient time (Figure S4). Drifts in *m/z* of single ions, related to the loss of small neutral or charged molecules, introduce significant bias in mass determination. The exact nature of such frequency drift events (e.g., loss of solvent molecules, metallic cations) might even be determined using a frequency-chasing approach.[45,46] This peak splitting phenomenon is enhanced for larger mRNAs that are harder to fully desolvate upstream within the mass analyzer. A simple but suboptimal way to avoid observing desolvation events is to acquire shorter transients (≤128 ms). Nonetheless, even if this approach may be enough to provide a first mass approximation of mRNA, decreasing the transient time comes at the cost of signal-to-noise (S/N), charge, and consequently, mass resolution. For mid-sized mRNAs that are lowly charged, reducing the transient length will be particularly detrimental for the signal intensity, as ions get closer to the noise threshold (Figure S5), making accurate ion tracing

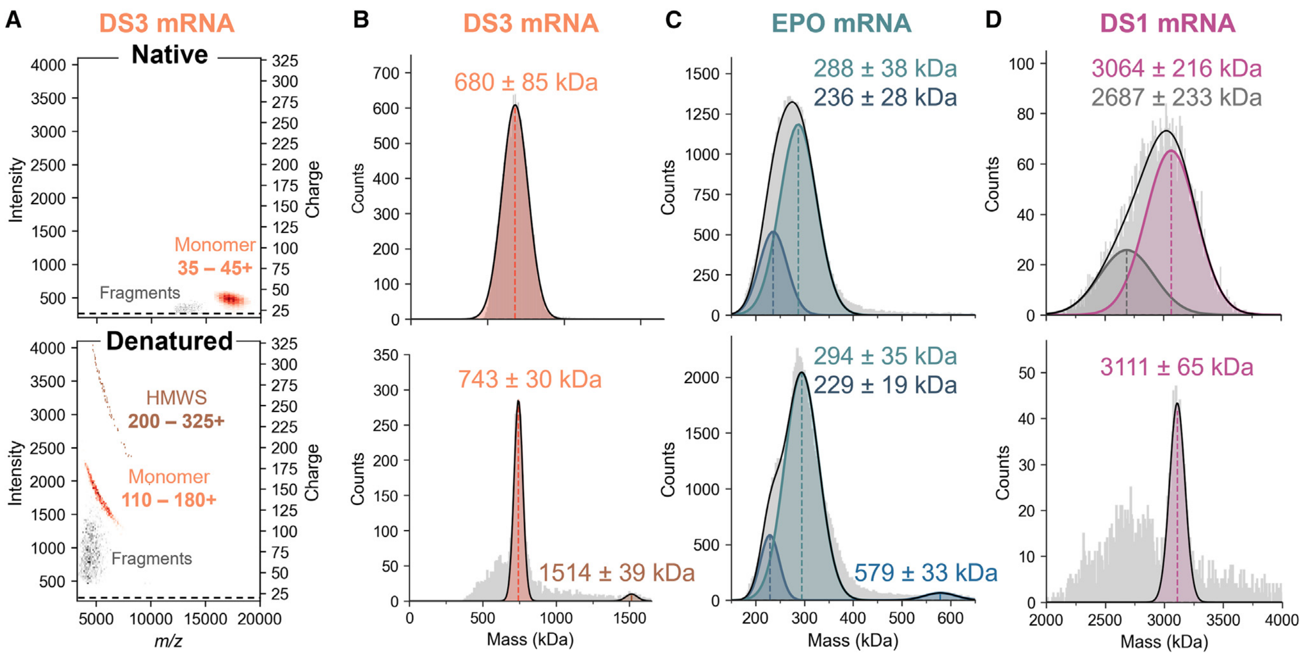

Figure 2. CDMS experiments performed on native and denatured populations of mRNA ions

Native and denatured populations were measured using 1:2 and 1:3 v/v $AcONH_4$/MeOH, respectively. Detailed experimental MS parameters are given in Table S1. (A) Two-dimensional histograms of single-ion signals for the DS3 mRNA. Native species (top) are close to the noise band, represented here as a black dashed line, due to their low charges. High charges originating from denaturation (bottom) are more intense in CDMS. (B) Mass histograms for the DS3 mRNA monomer and dimer, extracted from data shown in (A). Detected fragments have a mass of ∼400 kDa. (C) Mass histograms for the EPO mRNA revealing that at least two distinct mRNA species contribute to the main peak. (D) Mass histograms for the DS1 mRNA. All data obtained by native CDMS (low charges) are at top, and CDMS data under denaturing conditions (high charges) are at bottom.

more difficult. Larger mRNAs that are already impacted by the decreased resolving power at higher *m/z* and poor transmission will mostly suffer from the loss of charge and mass resolution.

Another avenue to circumvent the drawbacks of Orbitrap-based native CDMS for mRNA is to work on highly charged single ions, generated by the addition of methanol, which partially denatures mRNA (i.e., mRNA loses its native fold, as also seen using circular dichroism) (Figures 2A, S5 and S6). High charges directly translate into a charge error that is proportionally less impactful at low *m/z* compared to low charges in the high *m/z* range, and an increased S/N, which means that disadvantages of shorter transients are mitigated in denaturing CDMS of mRNA.

The DS3 mRNA example illustrates well the benefits of working on denatured ion populations instead of native ones. Two native, low-charged species corresponding to monomeric mRNA and fragments are detected close to the background level (Figure 2A). The mass extracted by CDMS for the single-stranded RNA is 680 ± 85 kDa, lower than the expected mass of 702 kDa (Figure 2B; Table 1). Due to partial denaturation, three unfolded populations are observed in low *m/z* regions, partially overlapping in *m/z* but separated in the charge dimension. The charge distribution for the denatured monomer spans from 110 to 180 charges, which is reflected in a higher S/N (Figure 2A). The mass resolution drastically improves, giving a mass of 743 ± 30 kDa, with the peak width in the mass histogram reduced by a factor of 3. Preliminary results recorded at extended transient lengths (24 s) show that the experimental mass eventually gets closer (704 kDa; Figure S7) to the theoretical mass (702 kDa), indicating that the initial mass excess at short transient times is mostly due to the presence of labile adducts and solvent molecules. Low amounts of species with twice the mass of the monomer (1,514 ± 39 kDa) are detected as well, and are also seen by an orthogonal capillary electrophoresis (i.e., fragment analysis) done under denaturing conditions (data not shown). Further investigation would be required to understand the nature of the observed dimer. Of note, experiments performed using isopropanol instead of methanol show a similar improvement in CDMS resolution, and the dimeric species is also observed (Figure S8).

Under denaturing conditions, mid-sized mRNAs like EPO (Figures 2C and S5) take advantage of the enhanced S/N, which facilitates ion tracing and data interpretation. For shorter mRNAs that are easier to desolvate prior to the Orbitrap, the mass is already close to the theoretical mass (283 kDa, Table 1) in native CDMS, but the denaturation helps to reveal a second species (229 ± 19 kDa) along with the main expected single-stranded RNA monomer (294 ± 35 kDa) and its dimer (579 ± 33 kDa). The gain in mass resolution is moderate for the

Table 1. Summary of theoretical masses for all mRNA products and masses measured by MP and denaturing CDMS

			CDMS (high charges)		MP	
Sample	Length, nt	Theoretical mass, kDa	Experimental mass, kDa	Deviation vs. theory, %	Experimental mass, kDa	Deviation vs. theory, %
EPO 5moU	859	282.9	288 ± 38	1.8	281 ± 56	−0.7
EGFP	997	323.5	327 ± 29	1.1	329 ± 54	1.7
Cre 5moU	1,351	444.4	451 ± 30	1.5	439 ± 55	−1.2
OVA	1,468	467.0	473 ± 44	1.3	459 ± 56	−1.7
Fluc	1,922	622.3	633 ± 35	1.7	624 ± 67	0.3
DS3 m1Ψ	2,162	702.2	743 ± 30	5.8	696 ± 61	−0.9
β-Gal	3,421	1,106.9	1,172 ± 44	5.9	1,092 ± 70	−1.3
DS1 m1Ψ	9,432	3,041.7	3,111 ± 65	2.3	3,050 ± 80	0.3
DS2 m1Ψ	9,472	3,054.9	3,174 ± 75	3.9	3,097 ± 80	1.4

Masses are reported for monomers only.

EPO mRNA compared to DS3 because resolution is still limited by the intrinsic sample heterogeneity and the transient length.

Perhaps the most striking improvement when working on highly charged ions is for mRNA in the higher megadalton range. Here, the mass obtained based on the native population is significantly lower than what is measured on denatured species (Figure 2D). In contrast to extended denatured ions, large and folded structures in their native state represent the worst-case scenario in terms of desolvation. Even if peak splitting is less pronounced at short transients, drifts in *m/z* due to desolvation can still occur within the first hundreds of milliseconds (Figure S4). In this case, the centroid value is shifted, and the intensity of the single ion is lower than expected because the signal intensity is already divided between at least two *m/z* peaks (i.e., peaks before and after neutral losses; see Figure S4C). This leads to an underestimation of the charge and subsequent mass. In addition, the charge accuracy deteriorates. However, at high charges, the impact on charge determination is reduced, and thus the mass resolution is substantially higher, enabling us to extract a mass of 3,111 ± 65 kDa for DS1, which corresponds to a deviation of 2.3% from the theory (Table 1).

Overall, our data show that recording short transients for the analysis of near-native, low-charge mRNA populations can hardly be used as a long-lasting solution in CDMS, as it requires substantial spray and MS optimization while offering only limited resolution and S/N performances. Working instead on highly charged ions improves mRNA mass determination for mid- to large mRNAs and enables high-resolution characterization of even very heterogeneous nucleic acids at the intact level.

MP for rapid mass and heterogeneity assessment of mRNA

As mRNAs proved to be challenging to analyze by MS, we additionally explored MP as a complementary single-particle approach. MP is a technique that determines the mass of a single molecule in solution from its scattering intensity upon landing on a glass slide.[47,48] As MP does not rely on any charge state assignment, the masses of extensively heterogeneous analytes can be measured. MP has previously been used for mRNA up to 1.5 MDa,[22,23] and we extend here its application to samples of several megadaltons, while offering additional details on how to handle MP measurements of mRNA as opposed to proteins.

Because mRNA molecules are negatively charged due to their phosphate backbone, they will not easily adhere to hydrophilic glass coverslips, contrary to proteins that bind to bare glass. Practically speaking, this translates into frequent unbinding events with positive contrast for mRNA (Figure 3A), making it impossible to measure their masses. To illustrate this behavior, we analyzed by MP a mixture of the protein complex immunoglobulin M (IgM) and Cre mRNA. On the standard glass cover slide, the mRNA molecules do not land properly. To detect the mRNA molecules, a more positive surface charge is thus mandatory. Therefore, we opted to functionalize the glass coverslips chemically by silanization using 3-aminopropyltriethoxysilane (APTES; see materials and methods). After APTES coating, both mRNA- (443 kDa) and protein- (950 kDa) binding events were successfully detected, with clearly varying molecule-to-molecule contrasts (Figure 3A).

Although we first intended to apply our standard protein-based calibration, we noticed that proteins and mRNA samples of similar sizes produced different contrasts (Figure S9). Calibrating nucleic acids using protein contrasts produced significant errors ranging from 7% to 10% compared to the theoretical masses. For example, the measured mass of the EPO mRNA is 256 ± 50 kDa, corresponding to a deviation of ∼9.5% from the expected value of 283 kDa (Table S2). These discrepancies can be explained by distinct refractive indices between the two types of analytes, which are alike as reported in MP for DNA molecules.[49,50] To calibrate for the DS1–3 samples, we constructed an in-house mRNA ladder using five samples from 996 to 3,420 nt. Our dataset demonstrated an excellent linearity of the contrast with the number of nucleotides on the whole mass range ($r^2 > 0.99$; Figure S9).

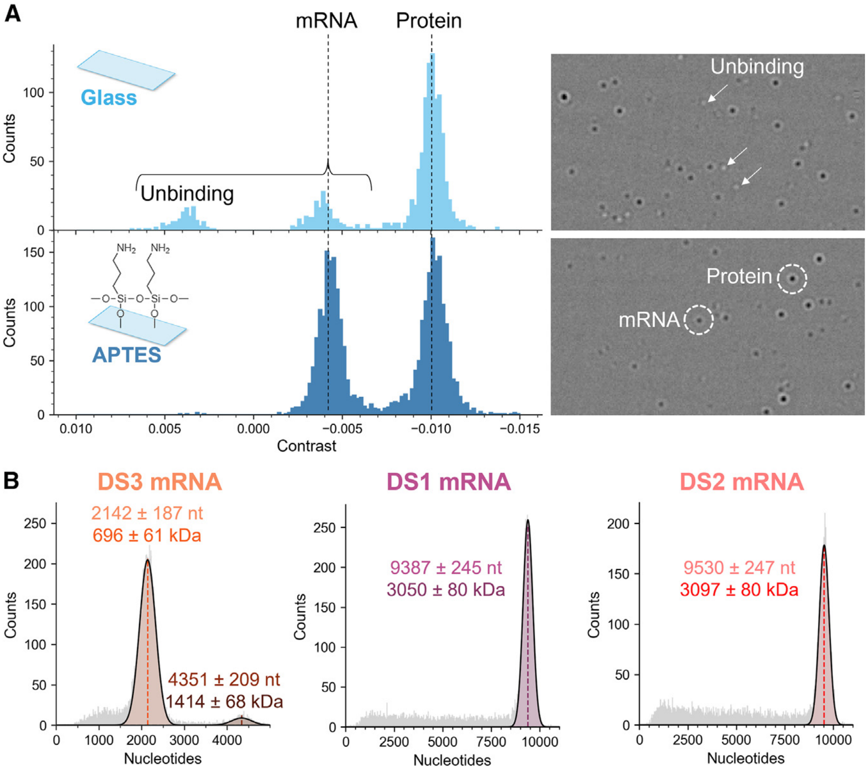

Figure 3. MP measurements of mRNA molecules
(A) Comparison of non-treated vs. APTES-coated glass slides for a mixture of a protein (IgM, 950 kDa) and an mRNA (Cre, 443 kDa) sample. Contrary to proteins, mRNA does not easily adhere to bare glass, producing unbinding events with positive contrasts, seen as white dots on the raw ratiometric image (right). APTES coating allows for mRNA binding, where only binding events (black dots) are detected. (B) Averaged (n = 3) histograms of nucleotide lengths for the three mRNA samples DS1, DS2, and DS3. The mass of each peak (centroid ± σ) was determined by Gaussian fitting. MP histograms of EPO, EGFP, Cre, OVA, Fluc, and β-Gal mRNA samples are shown in Figure S10.

With an mRNA-based calibration, differences <2% were obtained for mRNAs, which represents a clear improvement over the protein-based calibration (Figure S10; Table S2). Note that for commercial mRNAs, masses were calculated by successively considering each sample as unknown and not including it in the calibration curve. This proves that appropriate calibrants that match the nature of the analyte are mandatory for MP experiments.

Following appropriate mRNA calibration, the mass measured for the EPO mRNA (not included in the calibration curve) is 281 ± 56 kDa, corresponding to a deviation of 0.7% from the theory (Table 1; Figure S10). The smaller species seen in CDMS around 229 kDa are not observed here, mainly because the employed mass photometer does not provide enough resolution in the low mass range. When recorded on another mass photometer model offering higher resolution and more accurate quantification of low-molecular-weight species (LMWS), a left-side shoulder appears (Figure S11). This minor species, separated by ~42 kDa from the main EPO peak, could correspond to the monomer without its poly(A) tail (40 kDa), although further investigation by, for example, IP-RP or bottom-up approaches, would be required to confirm this hypothesis. Regarding the non-commercial mRNAs, the main species of DS3 could be accurately measured, with a mass of 696 ± 61 kDa, close to the theoretical mass of 702 kDa (Table 1). Low abundant mRNA fragments and traces of dimeric populations (1,414 ± 68 kDa) were detected (Figure 3B), as also seen by using CDMS. The proportion of dimer detected by MP (4.8%) with respect to the monomer agrees well with the CDMS data (3.7%). Lastly, MP was able to detect and distinguish even extremely large mRNAs, with masses of 3,050 ± 80 kDa for DS1 and 3,097 ± 80 kDa for DS2 (Figure 3B). This corresponds to differences of 0.3% and 1.4%, compared to the theoretical mass values, respectively, which is remarkable in this mass range (Table 1). Of note, fragments, here ranging from 200 kDa to 3 MDa, were detected in both samples. LMWS are also observed in the commercial mRNAs (Figure S10).

Our data show that MP can be used as an accurate method to determine the mass of mRNAs, provided that the coverslips and calibration are adapted. Although providing less resolving power in mass than denaturing Orbitrap-based CDMS, MP provides a rapid overview of mRNA sample heterogeneity.

DISCUSSION

Here, we show how native MS, Orbitrap-based CDMS, and MP can contribute to improve the assessment of heterogeneity and mass of mRNA samples ranging from 858 to ~9,400 nt (3 MDa). Native MS is suitable to measure mid-sized mRNAs, up to 1 MDa, although achieving a stable spray with usable signal is challenging. Native MS reaches its limits for mRNAs in the megadalton range, which only give broad unresolved charge distribution due to their size and poly(A) tail heterogeneity. Conversely, Orbitrap-based CDMS is particularly adapted for these larger mRNA products. We recommend using partial denaturation of mRNA to avoid non-ideal ion behaviors seen in native conditions. This yields both an enhanced S/N and increased mass resolution. In parallel, MP can be used as an in-solution technique to determine the mass of mid- to large mRNAs, although with a reduced resolution compared to our denaturing Orbitrap-based CDMS strategy. Nonetheless, it should be noted that it is often difficult to find a fit-for-all MS method that would allow for optimal transmission and desolvation of all species from LMWS to high-molecular-weight species (HMWS). The addition of methanol will likely disrupt non-covalent HMWS species, and mRNA fragmentation may also occur during the ESI process. Collectively, this will

make accurate quantification of the different species more challenging by CDMS, while MP has an advantage over CDMS of reflecting in an unbiased manner the presence and amounts of both LMWS and HMWS in a sample.

It should be noted that for now, the accuracy of both CDMS and MP does not allow the determination of the quantification of 5′ capping efficiency or the tackling of the microheterogeneity of the poly(A) tail (only the presence or absence of the tail can be assessed). Currently, bottom-up approaches are more suitable to determine the exact identity of the mRNA, from its 5′ capping to its tail.[17,41] However, the techniques presented here provide important orthogonal data compared to liquid chromatography (LC)-MS, providing information on mRNA integrity and potential impurities. In addition, we have recently demonstrated that it is possible to acquire longer transients (up to 25 s) in Orbitrap-based CDMS,[46] which enhance the charge resolution and sensitivity, meaning there is still room to improve the accuracy of the CDMS measurement. Prolonged trapping times would be beneficial for mRNA analysis, as we expect even large megadalton samples to be fully desolvated after 25 s, eventually getting closer to the theoretical masses, also under native conditions. Recording longer transients will, however, require relying on newly developed algorithms able to properly trace drifting ions (frequency chasing,[45] misSTORI[51]), which is not trivial in the case of mRNA, which displays extreme frequency shifts.

Lastly, as a further development to achieve an accurate quantification of all species, it could be foreseeable to rely on SEC separation coupled to ultraviolet light. New columns with ultrawide pore sizes enable the analysis of larger RNAs.[21,24] The recently reported efficient coupling of SEC to CDMS[52,53] would not only allow the separation and quantification of LMWS and HWMS from single-stranded mRNA samples but also ease the mass determination of each individual species by simplifying the mass spectrum, along with an improvement in spray reproducibility.

Orbitrap-based CDMS and MP will undoubtedly be useful to characterize not only mRNA but potentially also lipid nanoparticles that are used in mRNA-based vaccines and can be decorated with, for example, antibodies for improved efficiency. The techniques described here are therefore also of great interest for analyzing these even more complex next-generation therapeutics, including vaccines, viruses, and highly glycosylated antibodies.

MATERIALS AND METHODS

mRNA samples

EPO (858 nt), EGFP (996 nt), Cre (1350 nt), ovalbumin (OVA; 1,437 nt), firefly luciferase (Fluc; 1929 nt), and β-Gal (3,420 nt) mRNA samples were purchased from TriLink BioTechnologies. All mRNAs were modified with 3′-poly(A) tail (average length of 120 nt) and 5′-CleanCap1 (TriLink BioTechnologies). EPO and Cre mRNAs were fully substituted by 5-methoxyuridine (5moU). DS1 (9,432 nt), DS2 (9,472 nt), and DS3 (2,162 nt) mRNA samples, each modified with *N*1-methylpseudouridine (m1Ψ), were produced and provided by Pfizer. Theoretical masses for the different mRNAs are indicated in Table 1.

Sample preparation for native MS and CDMS experiments

mRNA samples were buffer exchanged into 150 mM ammonium acetate (Sigma) at pH 7.0 using ultracentrifugation filters (6 cycles, Amicon, 100 kDa molecular weight cutoff, Merck). To enhance the electrospray signal, samples were diluted in methanol (1:2 v/v or 1:3 v/v to induce denatured populations for CDMS) just before injection into the mass spectrometer. Approximately 3 μL of each sample was loaded into in-house prepared gold-coated pulled capillaries for nanoESI.

Native MS experiments

Native MS data were recorded on a Q-Exactive UHMR Orbitrap mass spectrometer (Thermo Fisher Scientific). Experiments were performed using a source capillary temperature of 250°C and a collision energy between 80 and 115 V. The source direct current offset was set to 21 V. In-source trapping was enabled, with desolvation voltages ranging between −20 and −60 V. The ion transfer optics (injection flatapole, inter-flatapole lens, bent flatapole, and transfer multipole) were set to 8, 7, 6, and 5 V, respectively, for mRNAs shorter than 1,000 nt. These values were changed to 5, 5, 6, and 6 V for mRNAs larger than 1,000 nt. The transient time was fixed to 32 ms. The injection time was set to between 50 and 100 ms. The trapping gas pressure setting was 4 (ultra-high vacuum [UHV] = 2×10^{-10} mbar) or 5 (UHV = 3×10^{-10} mbar, for mRNAs larger than 1,000 nt). Nitrogen was used as collision gas. Mass deconvolution was performed using UniDec version 5.1.1. Masses are reported based on Gaussian fits (centroid ± σ).

CDMS data acquisition and processing

For CDMS experiments, MS parameters (e.g., injection time, pressure, voltages) were tuned carefully to achieve single ion regime. A detailed list of parameters used to achieve optimal transmission and survival of native or denatured populations is given for the different samples in Table S1. Orbitrap-based CDMS data were processed in Python, as described previously.[32] Only frequency-domain data (i.e., the final mass spectrum per scan) were used for single ion analysis. Single ion intensities were corrected for injection time normalization. SciPy was used for Gaussian fitting of mass histograms (centroid ± σ).[54]

Preparation of coated glass slides for MP

Microscope coverslips (24 × 50 mm; Paul Marienfeld GmbH) were incubated overnight in 100 mM sulfuric acid (Merck). The slides were rinsed consecutively with Milli-Q water, methanol (Biosolve Chimie SARL, high-performance LC grade), ethanol (Supelco EMSURE), methanol, and ethanol. Coverslips were then left for 1 h in a solution of ethanol containing 5% APTES (Sigma) for glass coating. The coated slides were rinsed twice with ethanol before incubation in 6% acetic acid (Merck) for 30 min. Afterward, slides were rinsed with Milli-Q water and kept overnight in methanol. Finally,

the coated coverslips were cleansed with water and dried with N_2 before placing CultureWell gaskets (Grace Biolabs) on each slide.

MP measurements

MP experiments were performed on a SamuxMP instrument (Refeyn), unless stated otherwise. On the SamuxMP, 12 μL PBS were first loaded into a sample well for focusing of the mass photometer. Then, 3 μL diluted sample were added and mixed into the PBS droplet directly before data acquisition. The final concentration of mRNA in the droplet was 2–4 nM. Binding events were recorded for 60 s at 100 fps using AcquireMP version 2023 R2 (Refeyn). Contrast values were converted into mass values using calibrants of known masses. Protein-based calibration was done using thyroglobulin multimers at 335, 670, 1,005 and 1,340 kDa (T9145, Sigma). The mRNA-based calibration includes all commercial mRNA samples previously detailed, except for EPO mRNA.

On the TwoMP instrument (Refeyn) used for Figure S11, 18 μL PBS were loaded into a sample well on a glass slide coated with poly-L-lysine. Then, 2 μL diluted sample were mixed into the PBS droplet prior to data acquisition. The final concentration of mRNA in the droplet was 2–4 nM. Binding events were recorded for 60 s using AcquireMP. The mRNA-based calibration was performed using Invitrogen Millennium RNA Markers (Thermo Fisher Scientific), and the data were analyzed using DiscoverMP, in a similar way as the SamuxMP measurements.

Experimental mRNA lengths were determined in DiscoverMP version 2024 R1 (Refeyn) and exported for further processing by in-house Python scripts. SciPy was used for Gaussian fitting of mass histograms (centroid ± σ).

DATA AVAILABILITY

Data are available upon request from the authors.

ACKNOWLEDGMENTS

This research received funding from the Netherlands Organisation for Scientific Research (NWO) through the Spinoza Award SPI.2017.028 to A.J.R.H. E.D. and A.J.R.H. received financial support from Pfizer Inc. for this research.

AUTHOR CONTRIBUTIONS

A.J.R.H., T.W.P., and O.V.F. conceived the project. A.J.R.H. supervised the study. E.D. and L.F.B. performed the experiments. E.D. analyzed the data. E.D. and A.J.R.H. drafted the manuscript. All authors assisted with critical review and revisions of the manuscript.

DECLARATION OF INTERESTS

L.F.B., T.W.P., and O.V.F. are employees of Pfizer, the company that produced the DS1-3 mRNA samples studied here.

SUPPLEMENTAL INFORMATION

Supplemental information can be found online at https://doi.org/10.1016/j.omtm.2025.101454.

REFERENCES

1. Lee, I.T., Nachbagauer, R., Ensz, D., Schwartz, H., Carmona, L., Schaefers, K., Avanesov, A., Stadlbauer, D., Henry, C., Chen, R., et al. (2023). Safety and immunogenicity of a phase 1/2 randomized clinical trial of a quadrivalent, mRNA-based seasonal influenza vaccine (mRNA-1010) in healthy adults: interim analysis. Nat. Commun. *14*, 3631.
2. Xie, Z., Lin, Y.-C., Steichen, J.M., Ozorowski, G., Kratochvil, S., Ray, R., Torres, J.L., Liguori, A., Kalyuzhniy, O., Wang, X., et al. (2024). mRNA-LNP HIV-1 trimer boosters elicit precursors to broad neutralizing antibodies. Science *384*, eadk0582.
3. Weber, J.S., Carlino, M.S., Khattak, A., Meniawy, T., Ansstas, G., Taylor, M.H., Kim, K.B., McKean, M., Long, G.V., Sullivan, R.J., et al. (2024). Individualised neoantigen therapy mRNA-4157 (V940) plus pembrolizumab versus pembrolizumab monotherapy in resected melanoma (KEYNOTE-942): a randomised, phase 2b study. Lancet *403*, 632–644.
4. Mendez-Gomez, H.R., DeVries, A., Castillo, P., von Roemeling, C., Qdaisat, S., Stover, B.D., Xie, C., Weidert, F., Zhao, C., Moor, R., et al. (2024). RNA aggregates harness the danger response for potent cancer immunotherapy. Cell *187*, 2521–2535.e21.
5. Rojas, L.A., Sethna, Z., Soares, K.C., Olcese, C., Pang, N., Patterson, E., Lihm, J., Ceglia, N., Guasp, P., Chu, A., et al. (2023). Personalized RNA neoantigen vaccines stimulate T cells in pancreatic cancer. Nature *618*, 144–150.
6. Koeberl, D., Schulze, A., Sondheimer, N., Lipshutz, G.S., Geberhiwot, T., Li, L., Saini, R., Luo, J., Sikirica, V., Jin, L., et al. (2024). Interim analyses of a first-in-human phase 1/2 mRNA trial for propionic acidaemia. Nature *628*, 872–877.
7. Wang, Y.-S., Kumari, M., Chen, G.-H., Hong, M.-H., Yuan, J.P.-Y., Tsai, J.-L., and Wu, H.-C. (2023). mRNA-based vaccines and therapeutics: an in-depth survey of current and upcoming clinical applications. J. Biomed. Sci. *30*, 84.
8. Chaudhary, N., Weissman, D., and Whitehead, K.A. (2021). mRNA vaccines for infectious diseases: principles, delivery and clinical translation. Nat. Rev. Drug Discov. *20*, 817–838.
9. Duan, Q., Hu, T., Zhu, Q., Jin, X., Chi, F., and Chen, X. (2022). How far are the new wave of mRNA drugs from us? mRNA product current perspective and future development. Front. Immunol. *13*, 974433.
10. Ouranidis, A., Vavilis, T., Mandala, E., Davidopoulou, C., Stamoula, E., Markopoulou, C.K., Karagianni, A., and Kachrimanis, K. (2021). mRNA Therapeutic Modalities Design, Formulation and Manufacturing under Pharma 4.0 Principles. Biomedicines *10*, 50.
11. Liu, T., Liang, Y., and Huang, L. (2021). Development and Delivery Systems of mRNA Vaccines. Front. Bioeng. Biotechnol. *9*, 718753.
12. Gallie, D.R. (1991). The cap and poly(A) tail function synergistically to regulate mRNA translational efficiency. Genes Dev. *5*, 2108–2116.
13. Cheng, F., Wang, Y., Bai, Y., Liang, Z., Mao, Q., Liu, D., Wu, X., and Xu, M. (2023). Research Advances on the Stability of mRNA Vaccines. Viruses *15*, 668.
14. Patel, H.K., Zhang, K., Utegg, R., Stephens, E., Salem, S., Welch, H., Grobe, S., Schlereth, J., Kuhn, A.N., Ryczek, J., et al. (2023). Characterization of BNT162b2 mRNA to Evaluate Risk of Off-Target Antigen Translation. J. Pharmacol. Sci. (Tokyo, Jpn.) *112*, 1364–1371.
15. Mu, X., and Hur, S. (2021). Immunogenicity of In Vitro-Transcribed RNA. Acc. Chem. Res. *54*, 4012–4023.
16. Karikó, K., Muramatsu, H., Ludwig, J., and Weissman, D. (2011). Generating the optimal mRNA for therapy: HPLC purification eliminates immune activation and improves translation of nucleoside-modified, protein-encoding mRNA. Nucleic Acids Res. *39*, e142.
17. Guimaraes, G.J., Kim, J., and Bartlett, M.G. (2024). Characterization of mRNA therapeutics. Mass Spectrom. Rev. *43*, 1066–1090.
18. Fekete, S., Doneanu, C., Addepalli, B., Gaye, M., Nguyen, J., Alden, B., Birdsall, R., Han, D., Isaac, G., and Lauber, M. (2023). Challenges and emerging trends in liquid chromatography-based analyses of mRNA pharmaceuticals. J. Pharm. Biomed. Anal. *224*, 115174.
19. Yamauchi, Y., Taoka, M., Nobe, Y., Izumikawa, K., Takahashi, N., Nakayama, H., and Isobe, T. (2013). Denaturing reversed phase liquid chromatographic separation of non-coding ribonucleic acids on macro-porous polystyrene-divinylbenzene resins. J. Chromatogr. A *1312*, 87–92.
20. Currie, J., Dahlberg, J.R., Lundberg, E., Thunberg, L., Eriksson, J., Schweikart, F., Nilsson, G.A., and Örnskov, E. (2024). Stability indicating ion-pair reversed-phase

liquid chromatography method for modified mRNA. J. Pharm. Biomed. Anal. *245*, 116144.

21. Goyon, A., Tang, S., Fekete, S., Nguyen, D., Hofmann, K., Wang, S., Shatz-Binder, W., Fernandez, K.I., Hecht, E.S., Lauber, M., and Zhang, K. (2023). Separation of Plasmid DNA Topological Forms, Messenger RNA, and Lipid Nanoparticle Aggregates Using an Ultrawide Pore Size Exclusion Chromatography Column. Anal. Chem. *95*, 15017–15024.
22. Camperi, J., Lippold, S., Ayalew, L., Roper, B., Shao, S., Freund, E., Nissenbaum, A., Galan, C., Cao, Q., Yang, F., et al. (2024). Comprehensive Impurity Profiling of mRNA: Evaluating Current Technologies and Advanced Analytical Techniques. Anal. Chem. *96*, 3886–3897.
23. De Vos, J., Morreel, K., Alvarez, P., Vanluchene, H., Vankeirsbilck, R., Sandra, P., and Sandra, K. (2024). Evaluation of size-exclusion chromatography, multi-angle light scattering detection and mass photometry for the characterization of mRNA. J. Chromatogr. A *1719*, 464756.
24. D'Atri, V., Lardeux, H., Goyon, A., Imiołek, M., Fekete, S., Lauber, M., Zhang, K., and Guillarme, D. (2024). Optimizing Messenger RNA Analysis Using Ultra-Wide Pore Size Exclusion Chromatography Columns. Int. J. Mol. Sci. *25*, 6254.
25. Raffaele, J., Loughney, J.W., and Rustandi, R.R. (2022). Development of a microchip capillary electrophoresis method for determination of the purity and integrity of mRNA in lipid nanoparticle vaccines. Electrophoresis *43*, 1101–1106.
26. Vanhinsbergh, C.J., Criscuolo, A., Sutton, J.N., Murphy, K., Williamson, A.J.K., Cook, K., and Dickman, M.J. (2022). Characterization and Sequence Mapping of Large RNA and mRNA Therapeutics Using Mass Spectrometry. Anal. Chem. *94*, 7339–7349.
27. Jiang, T., Yu, N., Kim, J., Murgo, J.-R., Kissai, M., Ravichandran, K., Miracco, E.J., Presnyak, V., and Hua, S. (2019). Oligonucleotide Sequence Mapping of Large Therapeutic mRNAs via Parallel Ribonuclease Digestions and LC-MS/MS. Anal. Chem. *91*, 8500–8506.
28. Wolf, E.J., Grünberg, S., Dai, N., Chen, T.-H., Roy, B., Yigit, E., and Corrêa, I.R. (2022). Human RNase 4 improves mRNA sequence characterization by LC–MS/MS. Nucleic Acids Res. *50*, e106.
29. Gau, B.C., Dawdy, A.W., Wang, H.L., Bare, B., Castaneda, C.H., Friese, O.V., Thompson, M.S., Lerch, T.F., Cirelli, D.J., and Rouse, J.C. (2023). Oligonucleotide mapping via mass spectrometry to enable comprehensive primary structure characterization of an mRNA vaccine against SARS-CoV-2. Sci. Rep. *13*, 9038.
30. Brophy, P., Shion, H., Doneanu, C.E., Yang, H., Draper, B., Botamanenko, D., Abbatiello, S., Jarell, A., Koza, S., Yu, Y.Q., et al. (2022). Characterization of intact mRNA using ion-pair reversed-phase time-of-flight MS, size exclusion chromatography multi-angle light scattering, and charge detection mass spectrometry. In Proceedings of 70th ASMS Conference On Mass Spectrometry And Allied Topics, Minneapolis. Poster TP405.
31. Kafader, J.O., Beu, S.C., Early, B.P., Melani, R.D., Durbin, K.R., Zabrouskov, V., Makarov, A.A., Maze, J.T., Shinholt, D.L., Yip, P.F., et al. (2019). STORI Plots Enable Accurate Tracking of Individual Ion Signals. J. Am. Soc. Mass Spectrom. *30*, 2200–2203.
32. Wörner, T.P., Snijder, J., Bennett, A., Agbandje-McKenna, M., Makarov, A.A., and Heck, A.J.R. (2020). Resolving heterogeneous macromolecular assemblies by Orbitrap-based single-particle charge detection mass spectrometry. Nat. Methods *17*, 395–398.
33. Jarrold, M.F. (2022). Applications of Charge Detection Mass Spectrometry in Molecular Biology and Biotechnology. Chem. Rev. *122*, 7415–7441.
34. Deslignière, E., Rolland, A., Ebberink, E.H.T.M., Yin, V., and Heck, A.J.R. (2023). Orbitrap-Based Mass and Charge Analysis of Single Molecules. Acc. Chem. Res. *56*, 1458–1468.
35. Fuerstenau, S.D., and Benner, W.H. (1995). Molecular weight determination of megadalton DNA electrospray ions using charge detection time-of-flight mass spectrometry. Rapid Commun. Mass Spectrom. *9*, 1528–1538.
36. Schultz, J.C., Hack, C.A., and Benner, W.H. (1998). Mass determination of megadalton-DNA electrospray ions using charge detection mass spectrometry. J. Am. Soc. Mass Spectrom. *9*, 305–313.
37. Miller, L.M., Draper, B.E., Barnes, L.F., Ofoegbu, P.C., and Jarrold, M.F. (2023). Analysis of Megadalton-Sized DNA by Charge Detection Mass Spectrometry: Entropic Trapping and Shearing in Nanoelectrospray. Anal. Chem. *95*, 8965–8973.
38. Foreman, D.J., Stiving, A.Q., Klein, D.R., VanAernum, Z.L., Li, X., and Schuessler, H. (2022). Negative Mode Charge Detection Mass Spectrometry: Mass Determination Of Intact MRNA. In Proceedings of 70th ASMS Conference On Mass Spectrometry And Allied Topics, Minneapolis.
39. Lai, S.-H., Tamara, S., and Heck, A.J.R. (2021). Single-particle mass analysis of intact ribosomes by mass photometry and Orbitrap-based charge detection mass spectrometry. iScience *24*, 103211.
40. Largy, E., König, A., Ghosh, A., Ghosh, D., Benabou, S., Rosu, F., and Gabelica, V. (2021). Mass Spectrometry of Nucleic Acid Noncovalent Complexes. Chem. Rev. *122*, 7720–7839.
41. Gilar, M., Doneanu, C., and Gaye, M.M. (2023). Liquid Chromatography Methods for Analysis of mRNA Poly(A) Tail Length and Heterogeneity. Anal. Chem. *95*, 14308–14316.
42. Pleiss, J.A., Derrick, M.L., and Uhlenbeck, O.C. (1998). T7 RNA polymerase produces 5′ end heterogeneity during in vitro transcription from certain templates. RNA *4*, 1313–1317.
43. Gholamalipour, Y., Karunanayake Mudiyanselage, A., and Martin, C.T. (2018). 3′ end additions by T7 RNA polymerase are RNA self-templated, distributive and diverse in character—RNA-Seq analyses. Nucleic Acids Res. *46*, 9253–9263.
44. Konermann, L. (2017). Addressing a Common Misconception: Ammonium Acetate as Neutral pH "Buffer" for Native Electrospray Mass Spectrometry. J. Am. Soc. Mass Spectrom. *28*, 1827–1835.
45. Wörner, T.P., Aizikov, K., Snijder, J., Fort, K.L., Makarov, A.A., and Heck, A.J.R. (2022). Frequency chasing of individual megadalton ions in an Orbitrap analyser improves precision of analysis in single-molecule mass spectrometry. Nat. Chem. *14*, 515–522.
46. Deslignière, E., Yin, V.C., Ebberink, E.H.T.M., Rolland, A.D., Barendregt, A., Wörner, T.P., Nagornov, K.O., Kozhinov, A.N., Fort, K.L., Tsybin, Y.O., et al. (2024). Ultralong Transients Enhance Sensitivity and Resolution in Orbitrap-based Single Ion Mass Spectrometry. Nat. Methods *21*, 619–622.
47. Sonn-Segev, A., Belacic, K., Bodrug, T., Young, G., VanderLinden, R.T., Schulman, B.A., Schimpf, J., Friedrich, T., Dip, P.V., Schwartz, T.U., et al. (2020). Quantifying the heterogeneity of macromolecular machines by mass photometry. Nat. Commun. *11*, 1772.
48. Soltermann, F., Foley, E.D.B., Pagnoni, V., Galpin, M., Benesch, J.L.P., Kukura, P., and Struwe, W.B. (2020). Quantifying Protein–Protein Interactions by Molecular Counting with Mass Photometry. Angew. Chem. *132*, 10866–10871.
49. Li, Y., Struwe, W.B., and Kukura, P. (2020). Single molecule mass photometry of nucleic acids. Nucleic Acids Res. *48*, e97.
50. Kissling, V.M., Reginato, G., Bianco, E., Kasaciunaite, K., Tilma, J., Cereghetti, G., Schindler, N., Lee, S.S., Guérois, R., Luke, B., et al. (2022). Mre11-Rad50 oligomerization promotes DNA double-strand break repair. Nat. Commun. *13*, 2374.
51. Goodwin, M.P., Grinfeld, D., Yip, P., Bowen, K.P., Kafader, J.O., Kelleher, N.L., and Senko, M.W. (2024). Improved Signal Processing for Mass Shifting Ions in Charge Detection Mass Spectrometry. J. Am. Soc. Mass Spectrom. *35*, 658–662.
52. Strasser, L., Füssl, F., Morgan, T.E., Carillo, S., and Bones, J. (2023). Exploring Charge-Detection Mass Spectrometry on Chromatographic Time Scales. Anal. Chem. *95*, 15118–15124.
53. Yin, V., Deslignière, E., Mokiem, N., Gazi, I., Lood, R., de Haas, C.J.C., Rooijakkers, S.H.M., and Heck, A.J.R. (2024). Not All Arms of IgM Are Equal: Following Hinge-Directed Cleavage by Online Native SEC-Orbitrap-Based CDMS. J. Am. Soc. Mass Spectrom. *35*, 1320–1329.
54. Virtanen, P., Gommers, R., Oliphant, T.E., Haberland, M., Reddy, T., Cournapeau, D., Burovski, E., Peterson, P., Weckesser, W., Bright, J., et al. (2020). SciPy 1.0: fundamental algorithms for scientific computing in Python. Nat. Methods *17*, 261–272.

OMTM, Volume *33*

Supplemental information

Characterization of intact mRNA-based therapeutics by charge detection mass spectrometry and mass photometry

Evolène Deslignière, Lauren F. Barnes, Thomas W. Powers, Olga V. Friese, and Albert J.R. Heck

Table S1. Experimental parameters used for CDMS experiments under native and denaturing conditions. The parameters are given for mRNA samples reported in Figure 2. Intensity to charge calibration factors were calculated as described in Wörner, T.P. *et al.*, *Nat Methods* **2020**, 17, 395–398. Differences in calibration factors account for data being recorded on two different UHMR instruments.

		**EPO mRNA**	**DS3 mRNA**	**DS1 mRNA**	**EPO mRNA**	**DS3 mRNA**	**DS1 mRNA**
		Native (low charges)			***Denaturing (high charges)***		
**MS parameters**	Polarity	Positive	Positive	Positive	Positive	Positive	Positive
	Capillary voltage (kV)	1.5	1.5	1.5	1.5	1.5	1.5
	Capillary temperature (°C)	250	250	250	250	250	250
	S-lens RF level (%)	200	200	200	200	200	200
	m/z range	5,000-15,000	10,000-25,000	26,700-50,000	2,000-7,000	3,000-10,000	2,500-10,000
	Ion injection time (ms)	5	30	500	2	1	10
	Transient time (ms)	512	256	128	128	128	128
	Source DC offset (V)	21	21	21	-	21	21
	In-source trapping	Desolvation -40<br>Trapping 10	Desolvation -60<br>Trapping 10	Desolvation -70<br>Trapping 10	-	-	-
	HCD voltage (V)	120	115	110	50	60	60
	In-source CID (eV)	20	10	-	-	-	-
	Trap gas setting	4	3.5	3.5	2	3	2
	UHV readout (1e-10 mbar)	2.1	1.6	1.6	1.1	2	0.6
	Collision gas	Nitrogen	Nitrogen	Nitrogen	Nitrogen	Nitrogen	Nitrogen
	Injection flatapole (V)	8	8	4	8	8	8
	Inter-flatapole lens (V)	7	7	4	7	7	7
	Bent flatapole (V)	6	6	6	6	6	6
	Ion transfer target	High *m/z*	High *m/z*	High *m/z*	High *m/z*	High *m/z*	High *m/z*
	Detector optimization	High *m/z*	High *m/z*	High *m/z*	High *m/z*	High *m/z*	High *m/z*
	Intensity to charge calibration factor	12.52	14.07	14.07	14.07	12.52	14.07

Table S2. Overview of the experimental mRNA masses obtained in MP using a mRNA- *vs.* protein-based calibration. For mRNA data, each mass was calculated by building a new calibration curve that did not include the measured mRNA (i.e., by successively considering each mRNA as an unknown sample).

Sample	mRNA-based calibration		Protein-based calibration	
	Experimental mass (kDa)	Deviation from theory (%)	Experimental mass (kDa)	Deviation from theory (%)
**EPO 5moU**	281 ± 56	-0.7	256 ± 50	-9.5
**EGFP**	329 ± 54	1.7	290 ± 50	-10.4
**Cre 5moU**	439 ± 55	-1.2	399 ± 49	-10.2
**OVA**	459 ± 56	-1.7	418 ± 51	-10.5
**Fluc**	624 ± 67	0.3	577 ± 58	-7.3
**β-Gal**	1092 ± 70	-1.3	998 ± 67	-9.8

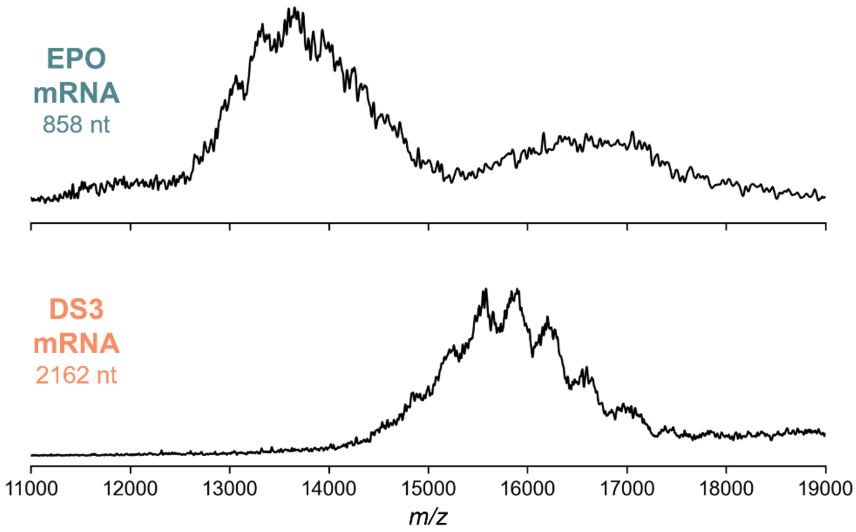

Figure S1. Native mass spectra of mRNAs recorded in negative ESI ion mode. Mass profiles exhibit more adducts than spectra acquired in positive ionization mode, making charge assignment and thus mass determination more difficult.

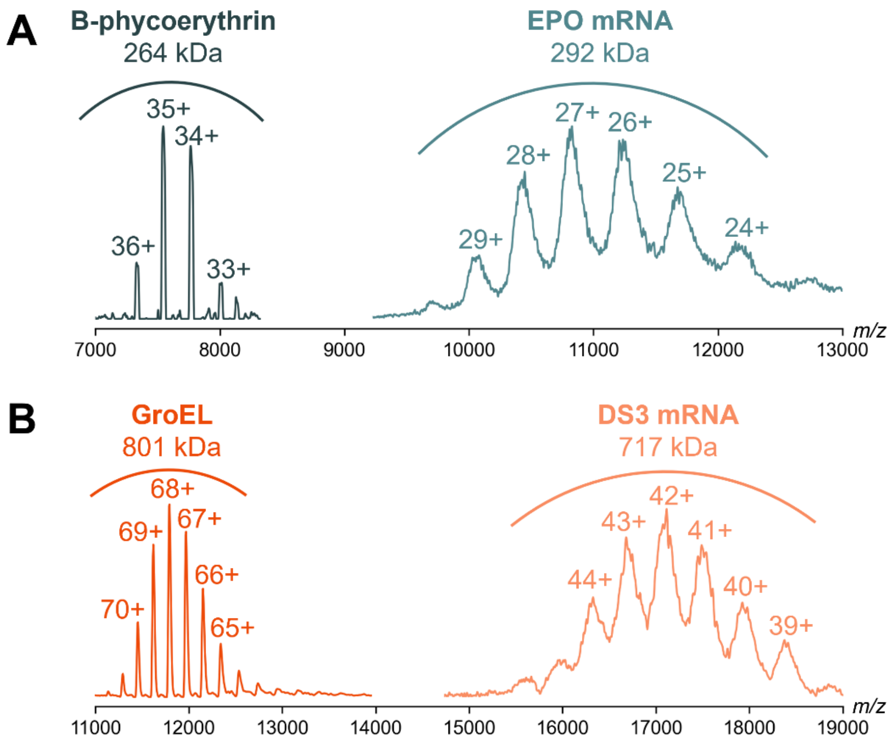

Figure S2. Native mass spectra of mRNA *vs.* proteins of similar sizes. Comparisons between **(A)** the protein complex B-phycoerythrin and EPO mRNA, and **(B)** the GroEL protein complex and DS3 mRNA. Spectra were recorded in positive ion mode and from 150 mM aqueous ammonium acetate solution. Note that mRNAs were sprayed by adding 50% methanol for better ionization efficiency.

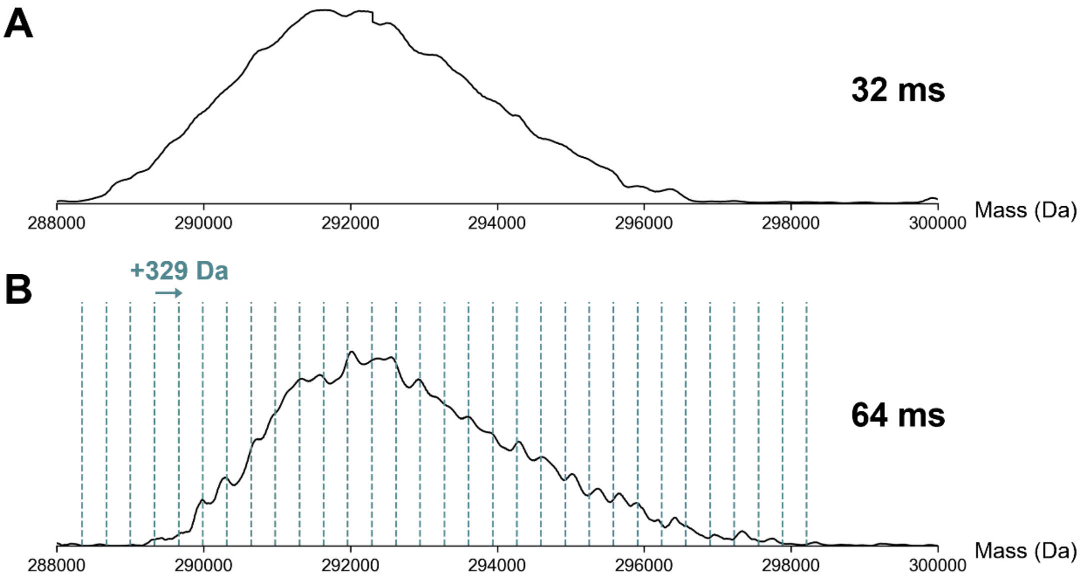

Figure S3. Mass deconvolution of EPO mRNA. Native mass spectra were recorded at transient lengths of **(A)** 32 ms and **(B)** 64 ms. The broad heterogeneous mass distribution is mostly due to the presence of poly(A) tail variants, separated by +329 Da (adenosine) as seen at extended transient length.

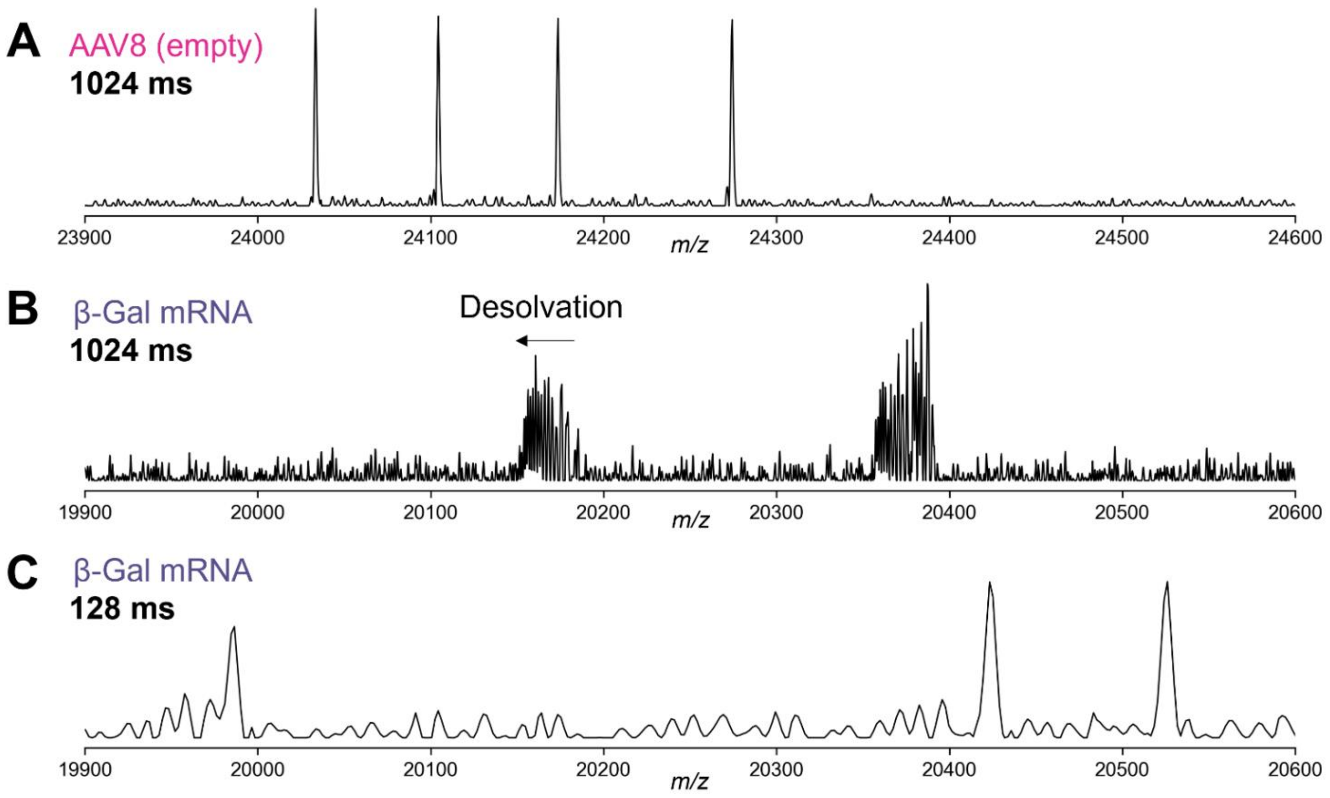

Figure S4. Non-ideal behaviors of mRNA single ions chased by CDMS, under native conditions. (A) In CDMS, single ions from an empty adeno-associated viral capsid (i.e. made up from proteins) are stable for 1 s. **(B)** In contrast, ions from the β-Gal mRNA show non-ideal behaviors over the 1 s-transient. Ions are drifting in *m/z* due to extensive desolvation. **(C)** By recording in CDMS shorter transients (128 ms) for the β-Gal mRNA, the ion drift is somewhat reduced, but can still occur (e.g. the ion at ~20,000 *m/z* has a lower intensity than expected due to neutral losses leading to peak splitting). Shorter transients result in a much lower resolution.

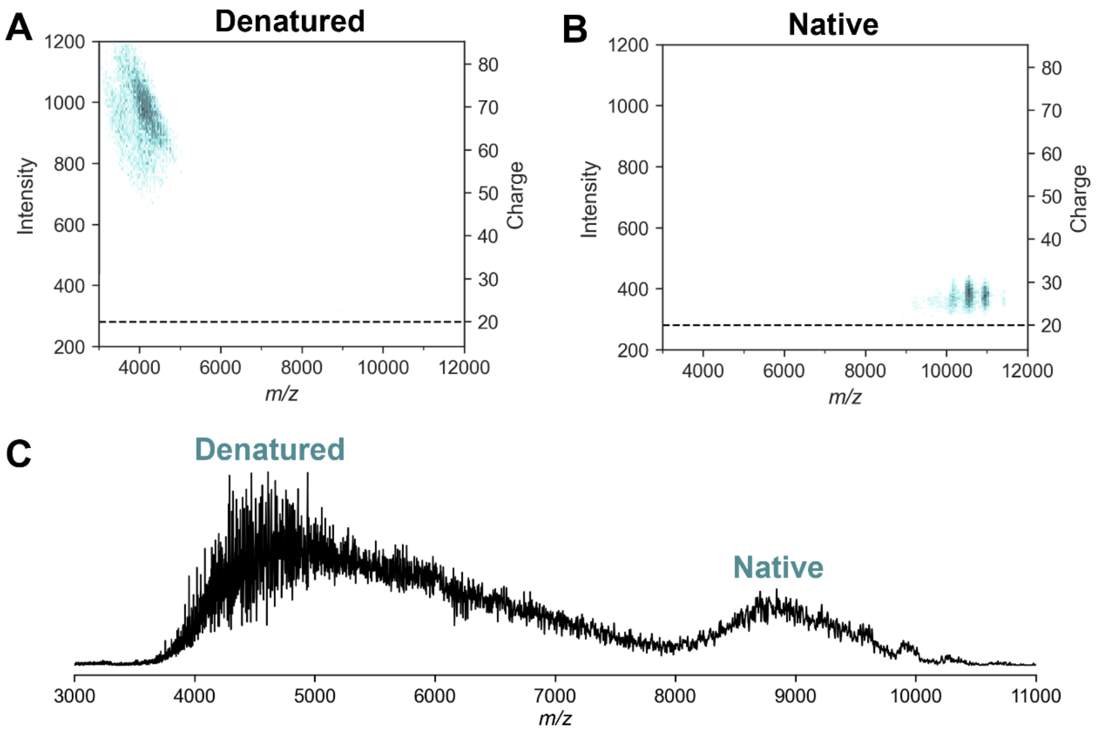

Figure S5. Native *vs.* denatured CDMS data for EPO mRNA. (A) Two-dimensional CDMS histogram of single ions in denaturing conditions (128 ms). The noise band is represented as a dotted line. **(B)** Two-dimensional CDMS histogram of native ions (512 ms). **(C)** Example of mass spectrum showing partial denaturation of the mRNA using methanol (1:3 v/v). MS parameters were tuned specifically to observe both populations simultaneously, and thus differ from those used in panels A and B.

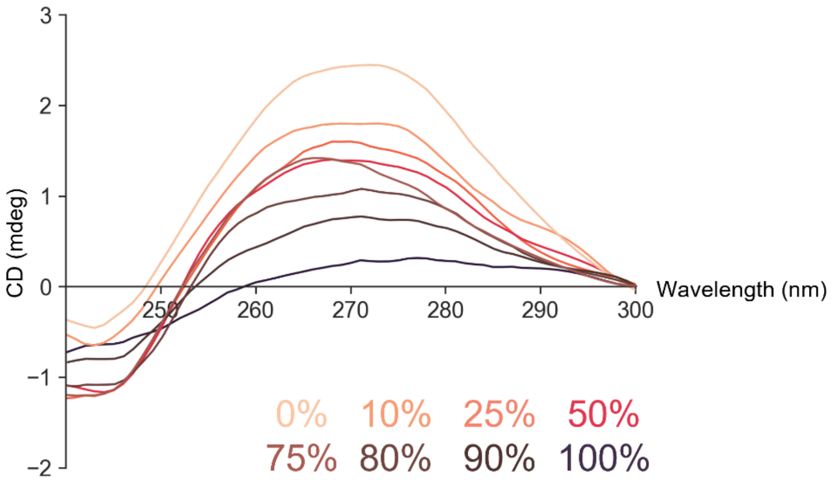

Figure S6. Circular dichroism spectra of the Cre mRNA, in water diluted with increasing percentages of methanol. Measurements were recorded in triplicate on a Jasco J-810 spectropolarimeter, using steps of 1 nm. For each solvent condition, the solvent-only spectrum (blank) was subtracted to the analyte measurement.

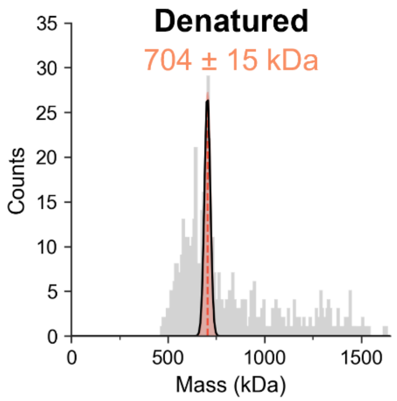

Figure S7. Mass histogram obtained by CDMS for the denatured DS3 mRNA using a transient length of 24 s. Extended transient lengths were recorded as reported in Deslignière, E. *et al.*, *Nat Methods* **2024**, 21, 619–622. The experimental mass at 24 s (704 kDa) is closer to the theory (702 kDa) than after 128 ms (743 kDa, Figure 2). This suggests that solvent molecules and labile adducts have been removed after prolonged trapping, and were contributing to the mass excess at short transients. Single ions undergo more collisions with neutral gas molecules as the transient time increases, with the mRNA eventually reaching full desolvation.

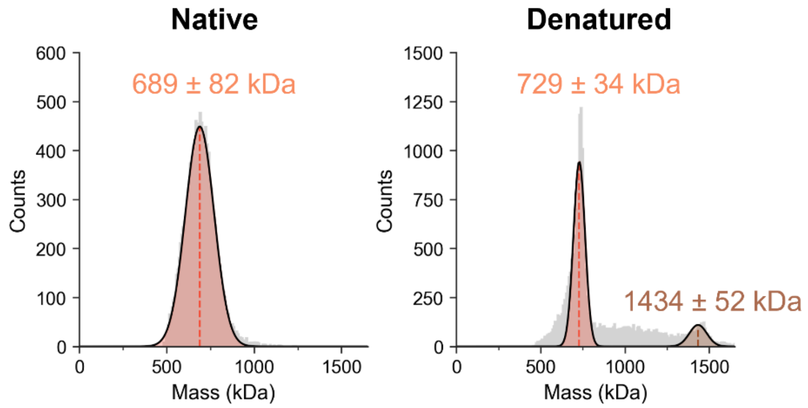

Figure S8. Mass histograms obtained by CDMS for the DS3 mRNA using isopropanol (1:3 v/v) instead of methanol to induce partial mRNA denaturation. Data were extracted from lowly charged species (37-50+, native) or highly charged populations (100-200+, denatured).

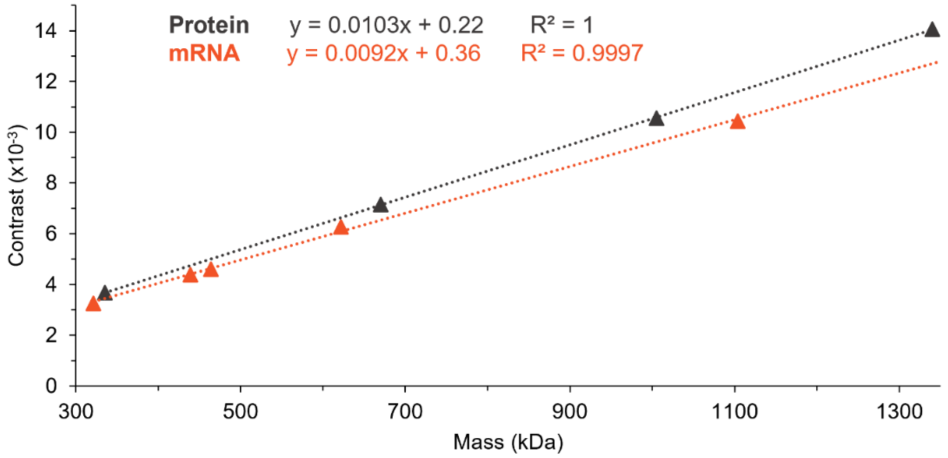

Figure S9. MP calibration curves using proteins (grey) or mRNAs (orange). The protein calibration is based on thyroglobulin multimers. The mRNA calibration contains EGFP, Cre, OVA, Fluc and β-Gal mRNAs. The differences in calibration curves show that the contrast highly depends on the nature of the analyte, with in particular large differences in between protein assemblies and mRNA.

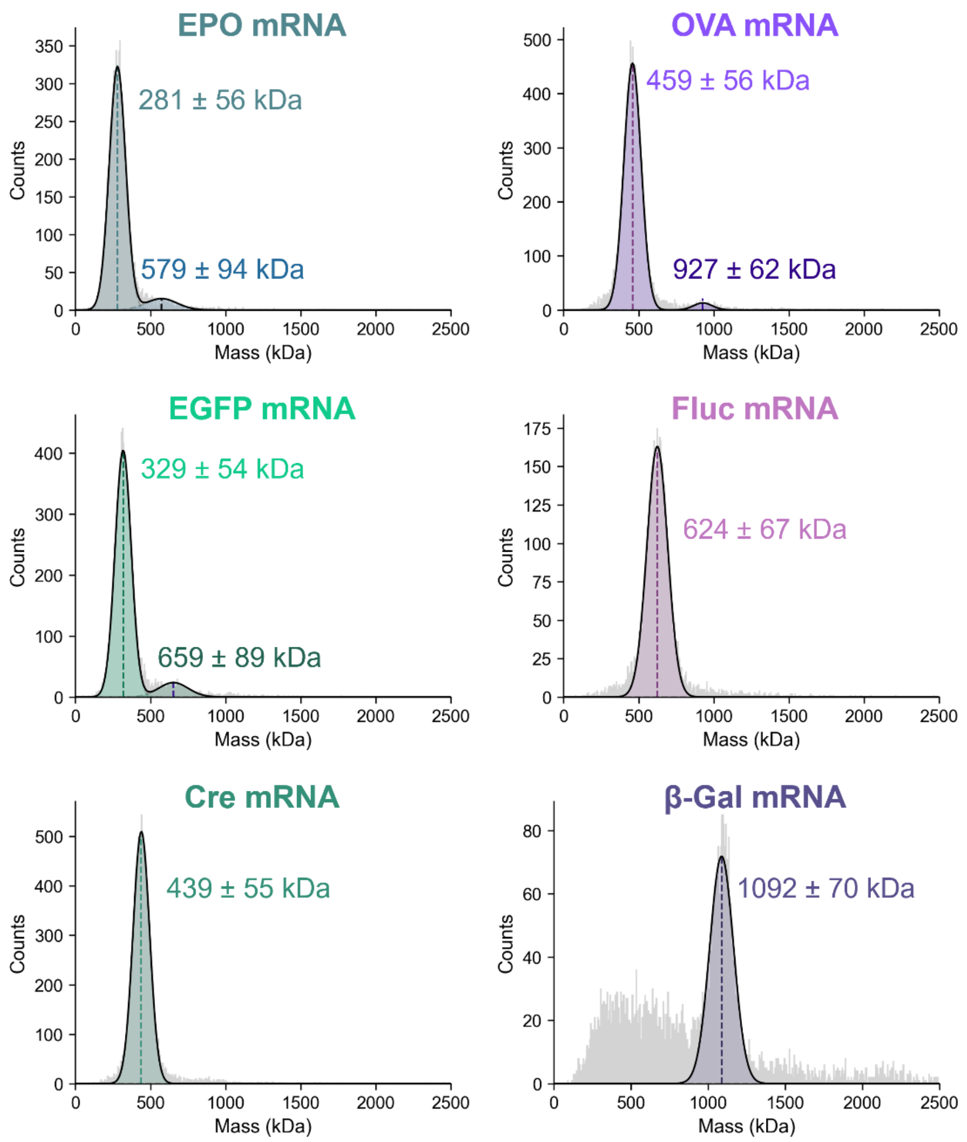

Figure S10. MP analyses of mRNAs. For all mRNAs, the mass of the main monomeric species is within 2% of the expected theory (see Table S2). Small amounts of dimeric populations are detected in EPO, OVA, and EGFP mRNAs. Many LMWS are clearly detected in the β-Gal sample, on the left of the main peak. Because the mass photometer used here is not adapted for the detection of LMWS < 250 kDa, it is likely that fragments are overlooked in other samples.

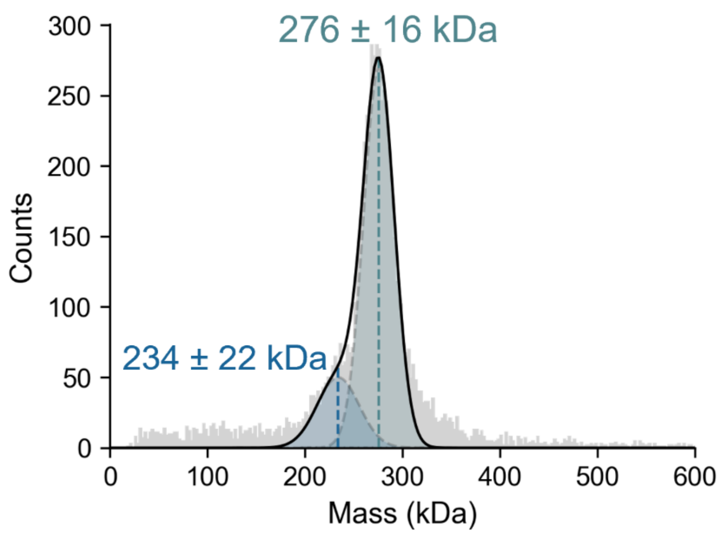

Figure S11. MP analysis of the EPO mRNA sample on a Two^{MP} mass photometer. This instrument is better adapted for the detection of LMWS < 250 kDa compared to the Samux^{MP}, the instrument used for the data described in the main manuscript. The Two^{MP} has a higher resolution, allowing to observe a left-side shoulder species of 234 kDa. Of note, dimeric EPO is not detected compared to what is seen on the Samux^{MP} (see Figure S10), which might be due to different glass slides coating used for the different experiments.
